# Elevated Air Humidity Changes Soil Bacterial Community Structure in the Silver Birch Stand

**DOI:** 10.3389/fmicb.2017.00557

**Published:** 2017-04-03

**Authors:** Marika Truu, Ivika Ostonen, Jens-Konrad Preem, Krista Lõhmus, Hiie Nõlvak, Teele Ligi, Katrin Rosenvald, Kaarin Parts, Priit Kupper, Jaak Truu

**Affiliations:** ^1^Department of Geography, Institute of Ecology and Earth Sciences, University of TartuTartu, Estonia; ^2^Department of Botany, Institute of Ecology and Earth Sciences, University of TartuTartu, Estonia

**Keywords:** air humidity, soil bacterial community, denitrifying bacteria, silver birch, root morphology

## Abstract

Soil microbes play a fundamental role in forest ecosystems and respond rapidly to changes in the environment. Simultaneously with the temperature increase the climate change scenarios also predict an intensified hydrological cycle for the Baltic Sea runoff region. The aim of this study was to assess the effect of elevated air humidity on the top soil microbial community structure of a silver birch (*Betula pendula* Roth.) stand by using a free air humidity manipulation facility (FAHM). The bacterial community structures of bulk soil and birch rhizosphere were analyzed using high-throughput sequencing of bacteria-specific16S rRNA gene fragments and quantification of denitrification related genes. The increased air humidity altered both bulk soil and rhizosphere bacterial community structures, and changes in the bacterial communities initiated by elevated air humidity were related to modified soil abiotic and biotic variables. Network analysis revealed that variation in soil bacterial community structural units is explained by altered abiotic conditions such as increased pH value in bulk soil, while in rhizosphere the change in absorptive root morphology had a higher effect. Among root morphological traits, the absorptive root diameter was strongest related to the bacterial community structure. The changes in bacterial community structures under elevated air humidity are associated with shifts in C, N, and P turnover as well as mineral weathering processes in soil. Increased air humidity decreased the *nir* and *nosZ* gene abundance in the rhizosphere bacterial community. The potential contribution of the denitrification to the N_2_O emission was not affected by the elevated air humidity in birch stand soil. In addition, the study revealed a strong link between the bacterial community structure, abundance of denitrification related genes, and birch absorptive root morphology in the ecosystem system adaptation to elevated air humidity.

## Introduction

Soil microbes play a fundamental role in forest ecosystems by stabilizing soil particles, performing organic matter decomposition, and mediating nutrient cycling and energy flow (Doran and Zeiss, 2000). Microbial activity is strongly dependent on nutritional, chemical, and physical conditions in the soil, and microorganisms respond rapidly to changes in soil properties caused by land management (Stark et al., 2007; Truu et al., 2009) and the climate (Grierson et al., 1999; Christopher et al., 2008). The global climate change is an unequivocal process (IPCC, 2007, 2013) that is accompanied by changes in the hydrological cycle (Held and Soden, 2006). A significant increase in precipitation, especially in northern regions (eastern parts of North America, Northern Europe, north, and central Asia) of the Earth, has been recorded for the period of 1900 to 2005 (IPCC, 2007). The global annual evapotranspiration has increased on average by 7.1 mm per year during the period of 1982 to 1997 (Jung et al., 2010), and the climate change scenarios predict further increase in air temperature (by 2.3–4.5°C) and in precipitation (by 5–30%) in Northern Europe for year 2100 (IPCC, 2007).

Climate change is driven by external forcing mechanisms (i.e., greenhouse gases) and natural variability of ecosystems (IPCC, 2007). Boreal forests are predicted to be more affected by the changed climatic conditions. An understanding of how these systems react to the changing environment is crucial for possible mitigation actions (IPCC, 2007; Poulter et al., 2013).

Since soil microbes (primarily prokaryotes and fungi) produce greenhouse gases, especially N_2_O (Seo and DeLaune, 2010; Giles et al., 2012; Saggar et al., 2013) and methane (Nazaries et al., 2013b), climate change-triggered alterations in soil microbial communities can have substantial feedback to the climate (Nazaries et al., 2013a). Denitrification is the main biological process causing N losses from forest soils (Fang et al., 2015; Morse et al., 2015). Depending on the set of denitrification genes possessed by soil microbial communities, the end product of this process can be either a harmless dinitrogen gas or a greenhouse gas (nitrous oxide–N_2_O). There are several processes that produce N_2_O, but the nitrous oxide reductase *NosZ* is the only enzyme known to catalyse the reduction of this compound in the environment (Thomson et al., 2012).

Several experimental systems have been established to estimate the effects of rising temperature and/or increasing atmospheric CO_2_ concentration on the functioning of forest ecosystems (Norby et al., 1997; Luxmoore et al., 1998; Pepin and Körner, 2002). Soil warming has been shown to decrease the ability of the microbial community to sustain its biomass in temperate mountain forest soils (Schindlbacher et al., 2011). Allison and Treseder (2008) reported that simultaneous temperature increase (0.5°C) and decline in water content (22%) of soil had negative effects on microbial respiration and bacterial and fungal abundances in boreal forest soils. The impacts of changed climate on soil nitrogen transformation processes (N-mineralisation, nitrification and denitrification) and nitrogen availability in various ecosystems (including forests) have been shown in many studies (Landesman and Dighton, 2010; Lupon et al., 2015). The characteristics of the climate change and concordant effect on N-cycling can be different according to the region (Lupon et al., 2015). Gerten et al. (2008) showed that the magnitude of changes in carbon and water dynamics in response to changes in precipitation is determined by the degree of the ecosystem water limitation (ratio between atmospheric transitional demand and soil water supply) and seasonal timing of precipitation change (doubling or halving rather than altered frequency and intensity at constant annual amounts).

In 2006, a Free Air Humidity Manipulation Facility (FAHM) was established in the south-eastern part of Estonia in order to study the effect of increased air humidity predicted for the Baltic region on deciduous forest (silver birch and hybrid aspen) ecosystems (Kupper et al., 2011). Results from this experiment have shown that the increased air humidity induced diverse changes in the functional traits of trees including the soil-to-leaf water transport pathway, resources, and biomass allocation patterns (Tullus et al., 2012; Sellin et al., 2013; Rosenvald et al., 2014). Parts et al. (2013) detected several changes in silver birch absorptive root morphology as well as in the ectomycorrhizal colonization pattern, reflecting the adaptation mechanism of this tree species at elevated air humidity conditions. Studies have shown that increase in soil water content and temperature leads to increase in N_2_O emission and to higher soil respiration rates as a positive feedback response of increased microbial metabolism (Cai et al., 2016; Oertel et al., 2016). In case of increased air humidity both the soil properties and plant traits are changed but the knowledge about the impact of increased air humidity on soil microbial community structures and functioning at potentially affected regions is still missing.

We hypothesize that the increased air humidity induces changes in soil bacterial community structures and functioning and affects the relationships within the bacterial-tree-root system. The aims of this study were to evaluate the impact of increased air humidity on the structures of bulk soil and birch rhizosphere bacterial communities and their N_2_O production and reduction potential, and to explore the effect of elevated air humidity on the soil-root-bacterial interactions in silver birch forests growing in a boreal (Baltic) region.

## Materials and methods

### Experimental site description

The Free Air Humidity Manipulation Facility (FAHM) with an area of 2.7 ha was established on former agricultural land at Rõka village (58°24′N, 27°18′E), Järvselja Experimental Forest District, Estonia in the spring of 2006. The experimental site contained eight hexagonal shape areas (four humidified and four control plots) each within a plot of 14 m × 14 m (Supplementary Figure 1). All plots were surrounded by buffer zones, and one half of each plot was planted with silver birch (*Betula bendula* Roth.) and the other half with hybrid aspen (*Populus tremula* L. × *P. tremuloides* Michx.) seedlings. Two types of understory vegetation communities were created on both tree species subplots. An early successional community was created by sowing a grass species (*Phleum pratense* L.), which dominated in surrounding old fields, and a “forest community” by transplanting patches of forest understory vegetation from a nearby forest and clear-cut to the study plots. The soil at the site was a fertile Endogenic Mollic Planosol (WRB).

On silver birch plots, 1-year-old seedlings were planted with a density of 10,000 trees ha^−1^ and a distance of 1 m between trees. Humidification started in June 2008 and has been carried out through all the following growing seasons (from mid-April to October). Misting took place if the ambient relative air humidity dropped below 75% and the wind speed was <4 ms^−1^. The system enables an average of 7% (maximum 18%) increase in relative air humidity above the ambient level. The meteorological (precipitation, wind speed and direction, air temperature, and relative humidity) and soil physical conditions (temperature and water potential) were constantly monitored in experimental plots during the vegetation periods. The detailed description of the whole experimental system is given by Kupper et al. (2011). The meteorological data (means of the relative air humidity and air temperatures for the experimental years and vegetation periods) as well as the soil physical characteristics (the means of water potential and temperature in 15 cm depth for the experimental years and vegetation periods) characterizing each studied plot from the beginning of the humidification experiment up to the second sampling time in October 2011 are given in Supplementary Table 1. In addition, the precipitation data (means of the experimental years and vegetation periods) for the whole site (Estonian Meteorological and Hydrological Institute) are given in this table.

### Soil and roots sampling and analyses

Two control and humidified plots (C1 and C4 plots and H1 and H4 plots, respectively) planted with birch were chosen for the microbiological analyses (Supplementary Figure 1). One composite sample composed of 10 pooled top soil cores (10 × 10 × 10 cm) was taken with a scoop from understory vegetation type subplots of each treatment plot (two composite samples per plot and four samples) in October 2009 and October 2011. Plant roots were removed from the composite bulk soil samples. Birch roots with the soil tightly attached to the birch roots were collected separately (rhizosphere samples). The soil samples were divided into subsamples for chemical analyses (stored at +4°C) and for DNA analyses (stored at −20°C). The pH_HCl_, organic matter content (SOM), and Kjeldahl nitrogen (total N) content for the soil samples of the control and humidified plots at both sampling times were determined and ratios of the understory and birch leaf litter mass (u/b) for each of the study plots were assessed (Table 1).

**Table 1:** **Mean and standard deviations of the control (*n* = 4) and humidified (*n* = 4) plots bulk soils physico-chemical characteristics and the ratios of understory and birch leaf litter mass at both sampling years**.

**Treatment**	**pH**_**KCl**_		**totN (%)**		**SOM (%)**		**Understory/birch**	
	**2009**	**2011**	**2009**	**2011**	**2009**	**2011**	**2009**	**2011**
Control	4.30 ± 0.13	4.27 ± 0.09	0.14 ± 0.03	0.15 ± 0.03	2.9 ± 0.4	2.8 ± 0.4	2.2 ± 1.5	0.7 ± 0.1
Humidified	4.45 ± 0.09	4.48 ± 0.12	0.12 ± 0.00	0.13 ± 0.01	2.6 ± 0.2	2.5 ± 0.2	2.8 ± 1.3	0.5 ± 0.2

*totN, total nitrogen content; SOM, soil organic matter*.

Birch absorptive fine roots (first and second order roots) were collected from the same soil of each plot and morphological parameters were determined as described by Parts et al. (2013). The following absorptive root parameters were measured and calculated: length (TipL, mm), diameter (Dia, mm), and surface area (SA, mm^2^); mean absorptive root dry mass (TipW, mg), root tissue density (RTD, kgm^−3^), specific root area (SRA, m^2^ kg^−1^), specific root length (SRL, mg^−1^), and root tip frequency per length and mass unit (RTF_L_, no mm^−1^ and RTF_W_, no mg^−1^, respectively).

#### DNA extraction, amplification and sequencing

DNA was extracted from 0.25 g bulk soil and rhizosphere (roots and attached soil) with a PowerSoil DNA Isolation Kit (Mo Bio Laboratories, Carlsbad, CA, USA), generally following the kit producer's protocol with an exception in the homogenisation step that was carried out at 5,000 rpm for 20 s using Precellys®24 (Bertin Technologies, Montigny-le-Bretonneux, France). The quantity and quality of DNA extracts were determined spectrophotometrically using Infinite M200 (Tecan AG, Grödig, Austria). The extracted DNA was stored at −20°C until further analyses.

Taxonomic profiling of the soil microbial community was performed using Illumina®HiSeq 2000 sequencing combinatorial sequence-tagged PCR products. The L-V6 and R-V6 primers (Gloor et al., 2010) were used to amplify the bacteria-specific V6 hyper variable region of the 16S rRNA gene. The PCR reaction mixture for amplification of each sample contained a unique combination of primers; each primer had specific 6 bp long barcode sequence at the 5′ end (Parameswaran et al., 2007).

All PCR reactions were performed in a 20 μl reaction mixture using a Phusion Hot Start High Fidelity Polymerase (Thermo Fisher Scientific, Waltham, MA, USA) according to the manufacturer's instructions. The DNA template concentration in the reaction mixture varied between 0.9 and 1.0 ng/μl. The amplification of each sample was performed in triplicate with the following touchdown PCR programme: initial denaturation at 98°C for 3 min, 6 thermal cycles of denaturation at 98°C for 5 s, annealing at 62°C for 30 s, decreasing by 1°C each cycle, and extension at 72°C for 10 s followed by 19 cycles of denaturation at 98°C for 5 s, annealing at 57°C for 30 s, and extension at 72°C for 10 s; the final extension step was performed at 72°C for 5 min. Replicate PCR products were pooled and the concentration of each composite sample was determined in a 2% agarose gel using a MassRuler® Express DNA Ladder (Thermo Fisher Scientific) and the Quantity One software (Bio-Rad Laboratories, Hercules, CA, USA). Amplicons from all samples were pooled in equal proportions and the mixture was purified and concentrated 4.5 times using the NucleoSpin® Extract II kit (Macherey-Nagel, Düren, Germany). Preparation of the paired-end DNA library was performed using the NEXTflexTM PCR-Free DNA Sequencing Kit (BIOO Scientific, Austin, TX, USA) and the sequencing was performed using the Illumina® HiSeq 2000 system (Illumina, San Diego, CA, USA).

#### Processing of sequenced dataset

The paired-end reads were assembled into composite reads with the PEAR programme (Zhang et al., 2013). In-house Perl scripts were used to sort sequences to the samples according to barcodes. The barcodes and primers were subsequently removed. The total initial number of sequences after assembling paired-end reads was 6,624,720 (8,064–511,904 reads per sample). All assembled reads were deposited in the European Nucleotide Archive under the accession number PRJEB9929. The assembled reads were analyzed using Mothur version 1.33.3 (Schloss et al., 2009) following modified standard operating procedure guidelines, with a notable difference being that the clustering step was carried out with the external programme, CROP (Hao et al., 2011). Sequences with low quality (containing ambiguous bases or more than six homopolymers, minimum read length of 70 bp, or an average sequencing quality score of <35 over a 25-bp sliding window) were discarded. In total, 6,530,818 usable reads were obtained (the total number of unique reads was 402,729). The remaining sequences were aligned to the SILVA-compatible reference alignment (Pruesse et al., 2007) to screen out overlapping sequences from resulting multiple sequence alignment for clustering. Suitable sequences (5,478,299 sequences—of which 101,957 sequences were unique) were clustered with CROP into operational taxonomic units (OTUs) at 95% similarity level. In the final step, the samples were normalized to the smallest sample size (6,086 reads) by random re-sampling in order to make them statistically comparable with each other. The taxonomic identity of each phylotype was determined with the Greengenes reference database (DeSantis et al., 2006).

#### Quantitative PCR analysis

The bacterial 16S rRNA gene copy numbers were quantified in bulk soil and rhizosphere samples using quantitative PCR (qPCR). The standard for total 16S rRNA gene enumeration was constructed as described by Nõlvak et al. (2012) using a fragment of *Pseudomonas mendocina* PC1 16S rRNA gene, which was amplified with the same primer pair, PCR mixture, and thermal conditions that were used for preparing PCR products for sequencing. Since the ratio between roots and soil was different in rhizosphere samples and the extracted amounts of target DNA were not comparable between samples, the obtained 16S rRNA gene qPCR data were used only for quantification of functional gene relative abundances in rhizosphere.

The structure of N_2_O producing (denitrifying) bacterial community in the bulk soil and rhizosphere was evaluated by the numbers of nitrite reductase-encoding *nirS* and *nirK* genes proportions (marked further as *nirS%* and *nirK%*, respectively) and the N_2_O production potential by the proportion of all *nir* genes (*nir%)* in the bacterial community. The N_2_O reducer community structure was analyzed using nitrous oxide reductase-encoding *nosZ* genes from clades I and II (marked as *nosZI%* and *nosZII%*, respectively) and N_2_O reduction potential using the proportion of all *nosZ* genes (*nosZ%*) in bulk soil and rhizosphere bacterial community. The primer pairs were nirScd3a and nirSR3d (Kandeler et al., 2006) for *nirS*, FlaCu and R3Cu (Hallin and Lindgren, 1999) for *nirK*, nosZF8 (Kloos et al., 2001) and nosZ1622R (Throbäck et al., 2004) for *nosZI*, and *nosZIIF* and *nosZIIR* (Jones et al., 2013) for *nosZII* amplification. The details about the primers and optimized qPCR reaction conditions are given in Supplementary Table 2. The qPCR assays were performed on the qPCR system Rotor-Gene®Q (Qiagen, Foster City, CA, USA). The optimized reaction mixture contained 5 μl Maxima SYBR Green Master Mix (Thermo Fisher Scientific), 1 μl of template DNA, 0.4–0.9 μM of reverse and forward primer, depending on the primer pair used (Supplementary Table 2) and sterile distilled water for a total volume of 10 μl. The optimized reaction conditions for most of the amplicons were: 10 min at 95°C; a sequence of 45 cycles of 15 s at 95°C, 30 s at the annealing temperature of the primer pair used (Supplementary Table 2), and 30 s at 72°C. The optimized reaction conditions for *nirS, nirK, nosZI*, and *nosZII* amplification included an additional extension step of 30 s at 80°C at the end of each cycle. In all cases, the fluorescence signal was read at the end of the final extension step of each cycle and a melting curve analysis was performed immediately after each qPCR assay. Triplicate qPCR reactions were run for each sample. The qPCR amplification efficiencies were evaluated using the LinRegPCR programme (version 2012.3). The 16S rRNA gene abundance in soil samples was calculated as an estimation of a fold difference between a sample and each 10-fold standard dilution in the range of 10^5^–10^8^ copies according to a formula and procedure proposed by Ruijter et al. (2009). 16S rRNA gene abundance in bulk soil is presented as gene copy numbers per a gram of dry soil (copies g^−1^ dw^−1^).

The qPCR data for denitrification genes were normalized against the bacterial 16S rRNA genes in order to calculate *nirS, nirK*, and *nosZI* and *nosZII*% in bulk and rhizosphere bacterial communities. The proportion calculations were performed using the calculation formula by Ruijter et al. (2009). The individual gene proportions were further used to calculate *nosZ*% by summing *nosZI* and *nosZII* and *nir*% by summing *nirS* and *nirK*%.

### Statistical analysis

The Pearson correlation coefficient was used to assess the relationships between the air and soil physical parameters during the vegetation periods of the study years. The *t*-test was applied to analyse the differences in these parameter values between C and H plots.

Diversity indices (Inverse Simpson diversity index - ISD) were calculated for bulk soil and rhizosphere samples. Principal component analyses (PCA) and redundancy analyses (RDA) with forward selection of variables were applied to explore and visualize differences between the studied soil groups, and to relate the soil physico-chemical characteristics and absorptive root morphology to soil bacterial community structure. Transformed values of OTU relative abundances that preserve Hellinger distance in ordination were used in PCA and RDA (Legendre and Gallagher, 2001). Canonical analysis of principal coordinates was applied to examine how variation in soil bacterial community composition was related to variation in environmental variables (Anderson and Willis, 2003). In addition, for comparative purpose ordination plots of principal coordinate analysis and nonmetric multidimensional scaling based on Bray-Curtis dissimilarity matrix were produced.

The effect of humidification, sampling time and the co-effect of these two factors on the bacterial community structure were evaluated by applying nested two-way permutational multivariate analysis (PERMANOVA) to Hellinger distance matrices (McArdle and Anderson, 2001). In order to detect differentially abundant OTUs between soils of C and H plots, linear discriminant analysis effect size (LEfSe) method was applied (Segata et al., 2011). LEfSe was run with parameter α for pairwise tests set to 0.05 for class normality, and the threshold on the logarithmic score of linear discriminant analysis was set to 2.0. This analysis was performed using only these OTUs that were classified at least on genus level (29% of all OTUs). In order to assess the species interactions within bacterial communities and evaluate the factors affecting different bacterial consortiums within community, phylogenetic molecular ecological networks (pMENs) were constructed using OTU data for bulk and rhizosphere soils by applying the Molecular Ecological Network Analyses Pipeline (MENAP; Deng et al., 2012). In order to test the differences between H and C plots, Hellinger distance matrices were obtained also for the pMENs modules and these distance matrixes were further used in nested PERMANOVA. PCA analysis was performed on each pMEN module, and the correlations between gene parameters and the first and second principal components were calculated. Spearman's rank correlation was applied to evaluate the relationships between gene parameters values and different environmental as well as birch root morphological parameters. In addition, the relationships between gene parameters' values and first two principal components of the pMEN modules and correlations between root morphological parameters and OTUs found to be affected by humidification via LefSe analyses was evaluated by the same method.

## Results

### Environmental conditions at the experimental plots

The meteorological data show that the climate conditions varied between experimental years at the site (Supplementary Table 1). Approximately two-times less precipitation was measured in the region during the vegetation period of year 2011, compared to the previous 3 years. Even though the onsite measurements showed higher relative air humidity (RH), soil water potential (SWP) and soil temperature (ST; differences on average of 1.5%, 36.2 kPa and 0.2°C, respectively), and about 0.1°C lower air temperature (AT) was measured in humidified (H) plots compared to the control (C) plots during all vegetation periods of the experimental years (Supplementary Table 1); however, the statistical analyses did not confirm the significance of these differences (*p* > 0.05 in all cases). The bulk soil pH varied between 4.16–4.46 in C plots, and 4.32–4.65 in H plots (Table 1). Statistical analyses revealed that pH was significantly higher (*t*-test, *p* < 0.01) in H plot soils at both sampling times. Soil total N content was between 0.12 and 0.17% and SOM content between 2.2 and 3.2% in all of the studied bulk soils and there were no significant differences between C and H plots in these parameter values. The ratio between understory and birch leaf litter mass was significantly decreased on both C and H plots in year 2011 compared to 2009 (*t*-test, *p* < 0.05), but no significant difference between treatments was revealed from the analysis.

### Bacterial 16S rRNA gene abundance and community structure

16S rRNA gene copy numbers ranged from 2.53 × 10^9^ copies g^−1^ dw^−1^ to 8.65 × 10^9^ copies g^−1^ dw^−1^in all studied bulk soils and the values were significantly higher in 2011 (*t*-test, *p* < 0.01) compared to 2009 (Supplementary Table 3). The gene copy numbers were much more variable in C soils and statistical analyses did not detect significant differences between C and H plots.

The analyses of the 16S rRNA gene fragments resulted from 324 to 425 OTUs per soil sample (Table 2). The obtained OTU numbers were significantly different (*t*-test, *p* < 0.05) between two study years only in bulk soils but did not differ between C and H plots. Bacterial diversity indices (ISD) were 1.1- to 1.5-times higher in bulk soils compared to rhizospheres. In addition, higher ISD values were obtained for bulk soils in 2011 than in 2009 (*t*-test, *p* < 0.05) and the difference between years was more pronounced in H plots (*t*-test, *p* < 0.01). The ISD values calculated for rhizosphere bacterial communities were not significantly different between C and H plots.

**Table 2:** **Mean and standard deviations of OTU numbers and diversity indices obtained for bulk soils and rhizospheres of the control (C; *n* = 4) and humidified (H; *n* = 4) plots at both sampling times**.

**Soil compartment**	**OTU number**				**Inverse Simpson index**			
	**2009**		**2011**		**2009**		**2011**	
	**C**	**H**	**C**	**H**	**C**	**H**	**C**	**H**
Bulk soil	387 ± 18	390 ± 13	408 ± 10	402 ± 5	70.4 ± 2.6	58.5 ± 7.5	74.2 ± 2.2	68.8 ± 2.9
Rhizosphere	370 ± 29	386 ± 37	342 ± 17	360 ± 3	48.3 ± 14.6	54.8 ± 8.8	55.3 ± 5.6	49.8 ± 2.6

**Figure 1:**
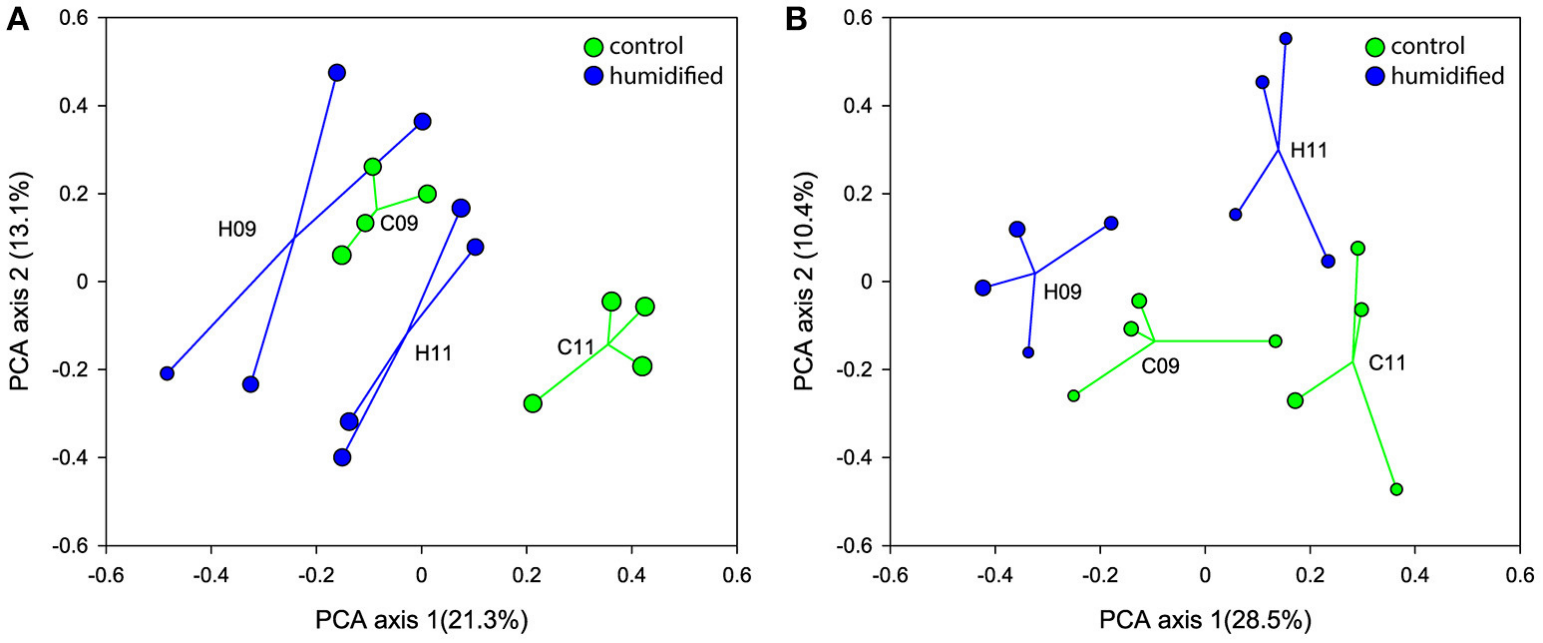
**Ordination of the bacterial communities of bulk soil (A)** and rhizosphere **(B)** samples according to principal component analysis that retains Hellinger distances between samples. The samples and centroids of the treatments (C–control plots and H–humidified plots in years 2009 (09) and 2011 (11)) are shown. The size of the sample circle is proportional to the Inverted Simpson index value.

The ordination of the bulk soil and rhizosphere samples, according to their bacterial community structure on the PCA plots, show shifts within 2 years in the bacterial community of both soil fractions at different air humidity conditions (Figures 1A,B, respectively, Supplementary Figure 2). In addition, different responses of microbial communities to the changed environmental conditions were revealed for bulk soil and rhizosphere. In 2009, the centroids of C and H plots were placed more closely on the PCA plot, whereas in 2011 they were more distant from each other due to the considerably larger changes in C plots in the case of bulk soils, and H plots in the case of rhizospheres.

Across all the soil samples, the most frequently found bacterial phyla were *Proteobacteria* (45.3–54.5%), *Actinobacteria* (11.6–19.8%), *Acidobacteria* (6.3–12.3%), *Bacteroidetes* (3.3–7.3%), and *Verrucomicrobia* (3.4–5.1%), but the proportions of representatives belonging to these phyla were different between bulk soils and rhizospheres. The proportions of *Proteobacteria, Actinobacteria*, and *Bacteroidetes* were larger in rhizospheres, while *Acidobacteria* and *Verrucomicrobia* were more abundantly represented in bulk soil bacterial communities (Supplementary Figure 3). The differences between bulk soil and rhizosphere were also revealed in the proportions of proteobacterial classes—*Alphaproteobacteria* dominated in rhizosphere, while *Betaproteobacteria* were the most abundant group in bulk soil. In addition, the bulk soil communities had several bacterial phyla (*Gemmatimonadetes, Planctomycetes, Chloroflexi*, and *Firmicutes*) that were not found among the dominating bacterial phyla in rhizospheres (except *Planctomycetes* and *Chloroflexi* in H plots in 2009). The proportion of phyla that were represented in lower numbers (<1%) in the bacterial community was between 2.62 and 3.54% in all studied soil groups and these groups contained representatives from 27 to 44 different phyla.

Results from nested PERMANOVA reveal a number of significant differences between two sampling times in the soil bacterial communities' structure of the plots at different organizational levels (whole community and dominant phyla) for both bulk soil and rhizosphere (Table 3). The analysis also revealed that the whole bacterial community structure as well as the structure of the three most dominant phyla was significantly different between C and H plots. In addition, the proportions of the dominant phyla were significantly reduced in H plots, but again the treatment effect on bacterial community was not similar in bulk soil and rhizosphere. The application of ANOVA on the proportions of proteobacterial classes revealed that *Beta*- and *Deltaproteobacteria* were more abundant, whereas *Gammaproteobacteria* were less abundant in H plots (of both bulk soil and rhizosphere) than in C plots (*p* < 0.001 and *p* < 0.05, *p* < 0.05, and *p* < 0.01, and *p* < 0.001 and *p* < 0.05, respectively) communities.

**Table 3:** Statistically significant differences (*n* = 8) based on permutation based nested ANOVA (PERMANOVA) between control and humidified plots, between two study years (time), and year and humidification interactions (Time^*^Treatment) in whole bacterial community, the three most abundant phyla and pMENs modules (marked with capital letters) structure detected in bulk soils and rhizospheres.

**Soil comp**.	**Community type/pMEN module**		**PERMANOVA**		
			**Time**	**Treatment**	Time^*^Treatment
Bulk soil	Whole		*p* < 0.001	*p* < 0.01	ns
	*Proteobacteria*		*p* < 0.001	*p* < 0.001	ns
	*Actinobacteria*		*p* < 0.001	*P* < 0.001	ns
	*Acidobacteria*		*p* < 0.01	*p* < 0.05	ns
	pMEN	A	*p* < 0.05	ns	ns
		B	*p* < 0.001	*p* < 0.001	*p* < 0.05
		C	*p* < 0.05	ns	ns
		D	*p* < 0.05	ns	ns
		G	*p* < 0.001	*p* < 0.01	*p* < 0.01
		H	*p* < 0.001	ns	ns
		I	*p* < 0.05	ns	ns
Rhizosphere	Whole		*p* < 0.001	*p* < 0.001	ns
	*Proteobacteria*		*p* < 0.001	*p* < 0.001	ns
	*Actinobacteria*		*p* < 0.01	*p* < 0.01	ns
	*Acidobacteria*		*p* < 0.01	ns	ns
	pMEN	J	*p* < 0.001	ns	ns
		K	*p* < 0.01	*p* < 0.01	ns
		L	*p* < 0.001	*p* < 0.05	ns
		N	*p* < 0.05	ns	ns
		O	*p* < 0.001	*p* < 0.05	ns
		P	*p* < 0.001	ns	ns
		Q	*p* < 0.01	ns	*p* < 0.05

*ns, not significant*.

**Figure 2:**
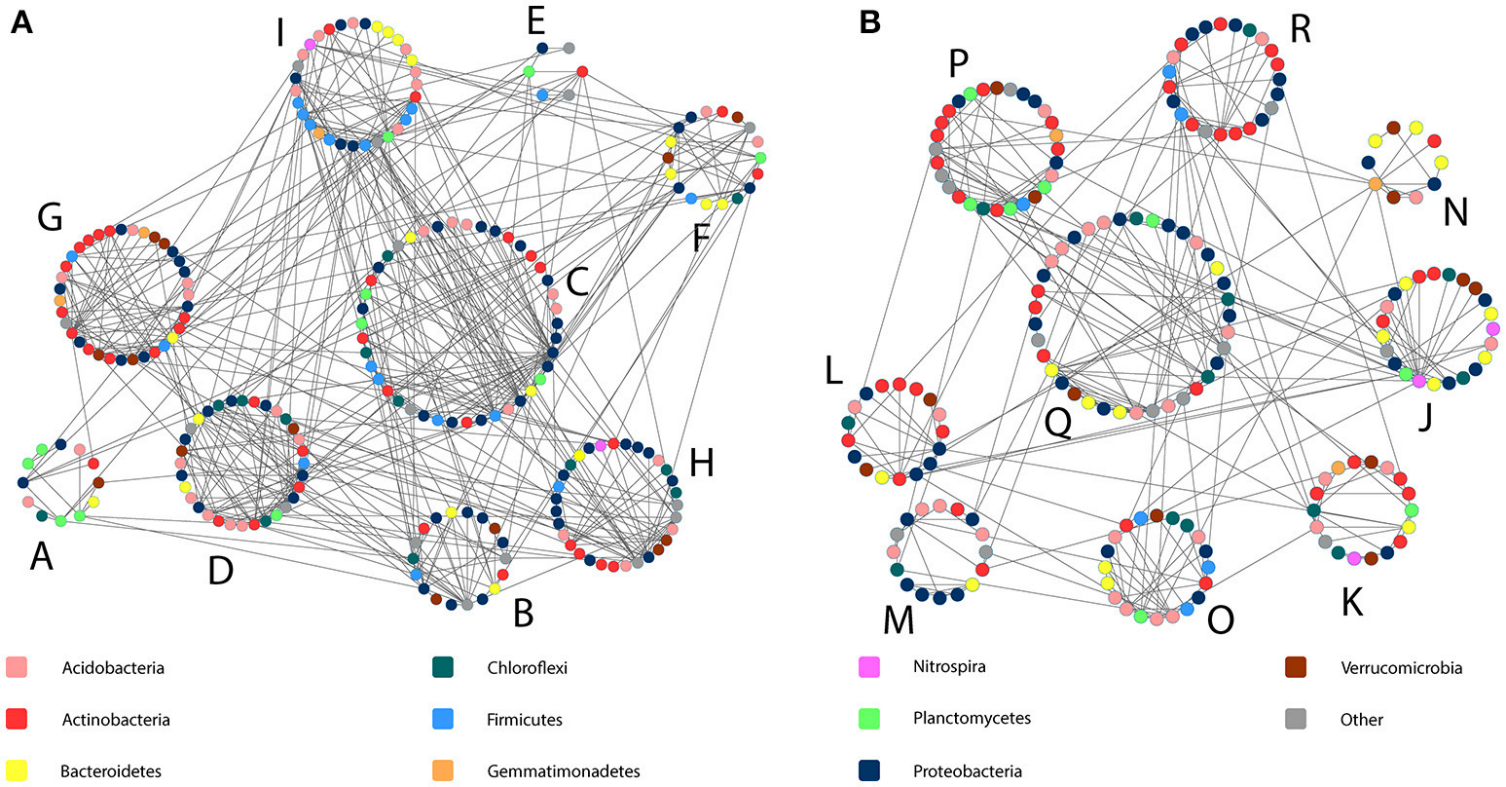
**Bacterial phylogenetic molecular ecological networks obtained for birch stand bulk soil and rhizosphere bacterial communities** (**A** and **B**, respectively). The network modules are indicated with capital letters and each colored spot within each module indicates one bacterial phylotype (OTU). Shown are only modules with more than five OTUs. The information on the taxonomy of OTUs is given in Supplementary Table 5.

As a result of the application of MENAP, phylogenetic molecular ecological networks (pMENs) with different topological properties were obtained for bulk soils and rhizospheres (Figures 2A,B, Supplementary Table 4). According to this analysis, 36 and 42% of the analyzed sequences were found to be involved in bulk soil and rhizosphere networks, respectively. A network with nine modules for both studied soil compartments was obtained where the modules eigen-gene values ranged from 21 to 38% and from 24 to 55% for bulk soil and rhizosphere pMEN, respectively. Each module in both pMENs had a unique bacterial phylotype composition (Supplementary Table 5). Nested PERMANOVA results show that there were significant structural differences between sampling years, also at the level of ecological network modules. But in addition, the analyses revealed two bulk soil modules (B and G) and four rhizosphere modules (K, L, O, and Q) where the proportions of OTUs were significantly different in C and H plots (Table 3). In the cases of the two bulk soil modules, the treatment effect was different in the two sampling times. The difference between C and H plots in the proportions of OTUs for rhizosphere module Q was detected (as a time and treatment interaction) in 2011.

The LEfSe analysis detected in total 27 phylotypes (4.7 and 5.1% from all the OTUs classified at genus level in bulk soils and rhizospheres, respectively) belonging to 23 bacterial genera whose proportions were significantly different between the C and H plots (Supplementary Tables 5, 6). When most of the affected phylotypes were found in the pMENs (in one or in both soil fractions), six of them were not involved in the networks (Supplementary Table 6). The modules possessing the highest numbers of affected OTUs were the bulk soil modules C and G (both with eight phylotypes) and rhizosphere modules P and M (both with four phylotypes). We did not find these OTUs from bulk soil modules D and E and from rhizosphere modules J, L, and N.

Proteobacterial genera were among the bacterial groups affected by humidification but the effect (positive or negative) depended on the phylotype. Only one phylotype (OTU49) from the genus *Geobacter* was more abundant in both bulk soil and rhizosphere of H plots while the other phylotypes varied between bulk soil and rhizosphere. In addition, the proportion of two phylotypes (from the genera *Rhodanobacter* and *Pseudomonas*) in both soil compartments of the C plots exceeded those of the H plots. Meanwhile, different phylotypes from the genus *Candidatus Solibacter* in bulk soils of H plots outnumbered C plots but at the same time were in the minority in rhizospheres of H plots. The proportions of different *Actinobacteria* genera were higher in C plots (in both soil fractions) than in H plots.

### Abundance of the denitrification genes in bacterial community

The quantification of denitrification specific genes revealed differences between bulk soil and rhizosphere in denitrifying bacterial community structure and its N_2_O production and reduction potential. When *nirS* and *nosZII*% were in a similar range in both studied soil fractions (3.99 ± 1.03% and 3.16 ± 1.58%, and 0.31 ± 0.11% and 0.33 ± 0.26% in bulk soils and rhizosphere, respectively) then *nirK* and *nosZI*% were significantly more abundant in rhizospheres (6.19 ± 1.27% and 9.45 ± 2.71%, and 0.59 ± 0.14% and 3.23 ± 1.28% in bulk soils and rhizospheres, respectively; *p* < 0.001 in both cases).

Significantly lower *nirK* and *nosZI*% were measured in rhizosphere of H plots (*t*-test, *p* < 0.001, and *p* < 0.05, respectively) but in the case of *nirK*% the effect was more pronounced in 2009 than in 2011 (*p* < 0.05). The humidification affected also rhizosphere *nir* and *nosZ%* whereby significantly lower parameter values for these were found for H plots compared to C plots (*p* < 0.05 in both cases, Supplementary Table 3).

All the analyzed denitrification gene ratios were different in the bulk soil and rhizosphere: *nirS/nirK, nosZII/nosZI*, and *nir/nosZ* were significantly higher in bulk soils (*p* < 0.001 in all cases) but only *nirS/nirK* was different between C and H rhizospheres (higher in H plots, *p* < 0.01).

Several denitrification gene proportions and their ratios were related to the first two principal components of the bulk soil and rhizosphere whole bacterial community as well as the pMEN modules (Table 4). Most of the pMEN modules (except the bulk soil modules D and F, and rhizosphere module Q) were related to the denitrification gene parameters. A number of relationships for *nirS%, nirS/nirK*, and *nir/nosZ* were detected in both soil fractions but there was a considerably higher number of significant relationships between community structure and total *nir* genes (*nir%*), as well as different *nosZ* gene parameters that were revealed for rhizosphere. *NirK%* was related only to rhizosphere bacterial community structure.

**Table 4:** **Significant Spearman correlations between the studied gene parameters and first two principal components of the whole bulk soil and rhizosphere bacterial communities and respective pMENs modules (indicated with capital letters)**.

**SC**	**CT**	**PC**	**Gene parameter**								
			***nirS%***	***nirK%***	***nirS/nirK***	***nir%***	***nosZI%***	***nosZII%***	***nosZII/nosZI***	***nosZ%***	***nir/nosZ***
Bulk soil	Whole	1	−0.54^*^		−0.79^***^						−0.59^*^
	A	1			−0.52^*^		0.50^*^				−0.61^*^
	B	1	−0.50^*^		−0.67^**^						−0.52^*^
	C	2									−0.55^*^
	E	1						−0.58^*^	−0.56^*^		
		2	−0.51^*^								
	G	1	−0.78^***^		−0.65^**^	−0.55^*^					
	H	1	−0.50^*^		−0.68^**^						
	I	1			−0.80^***^						−0.61^*^
Rhizosphere	Whole	1	−0.51^*^		−0.84^***^			−0.62^**^	−0.51^*^		
		2		−0.72^**^		−0.72^*^	−0.57^*^			−0.58^*^	
	J	1	−0.60^*^		−0.74^**^		−0.54^*^	−0.72^**^	−0.51^*^	−0.61^*^	0.61^*^
	K	1			0.68^**^						
		2		0.81^***^		0.75^**^	0.69^**^			0.69^**^	
	L	1			0.80^***^			0.54^*^			−0.59^*^
	M	1				0.53^*^					
		2					−0.55^*^			−0.56^*^	
	N	1			−0.53^*^		−0.61^*^			−0.60^*^	0.69^**^
	O	1	0.70^**^		0.66^**^		0.59^*^	0.78^***^	0.63^*^	0.67^**^	−0.52^*^
	P	1	−0.74^**^		−0.68^**^		−0.63^*^	−0.73^**^		−0.65^**^	0.64^**^
	R	1	−0.63^*^	−0.55^*^		−0.66^**^		−0.52^*^			

* p < 0.05;

** p < 0.01;

*** *p < 0.001*.

*SC, soil compartment; CT, bacterial community type; PC, principal component*.

In addition, the statistical analyses found significant relationships (Spearman correlations) between functional gene proportions in bacterial community and proportions of the bacterial phylotypes found to be different in H and C plots of rhizosphere soils, according to the LEfSe analyses. In bulk soils, the *Pedomicrobium* spp. was related to *nirK%* and *nirS/nirK* (*R* = −0.51, *p* < 0.05 and *R* = 0.60, *p* < 0.05, respectively). In rhizosphere, the phylotypes from the genera *Clostridium, Geobacter* (OTU49), *Ca. Xiphinematobacter, Ca. Solibacter, Streptacidiphilus*, and *Pseudomonas* (OTU779) were related to *nirK%* (*R* = −0.78, *p* < 0.001; *R* = −0.77, *p* < 0.001, *R* = −0.66, *p* < 0.01; *R* = 0.69, *p* < 0.01; *R* = 0.83, *p* < 0.001 and *R* = 0.71, *p* < 0.01, respectively). In addition, the phylotypes from genera *Ca. Xiphinematobacter, Luteolibacter, Acidobacterium*, and *Rhodanobacter* were related to *nirS/nirK* (*R* = 0.69, *p* < 0.01; *R* = 0.51, *p* < 0.05; *R* = −0.77, *p* < 0.001 and *R* = −0. 69, *p* < 0.01, respectively), *Acidobacterium* to *nosZII%* (*R* = −0.51, *p* < 0.05) and *Rhodanobacter* to *nir/nosZ* (*R* = 0.65, *p* < 0.01) in this soil fraction.

### Relationships between environmental factors and soil bacterial community structure

Canonical analysis of principal components (CAP) was applied to assess and visualize relationships between soil bacterial community structure and environmental variables (Supplementary Figures 4, 5). Both in case of bulk soil and rhizosphere samples the relationships with plot climatic and soil conditions were stronger than with root traits. Results from RDA analysis with forward selection showed that the whole bacterial community structure in both soil fractions was significantly affected by plot climatic conditions (RH and AT, Table 5). In addition, soil chemical composition (SOM content) had a significant effect on the whole rhizosphere bacterial community structure. The analyses also revealed that the structural units (pMEN modules) within the whole bacterial community were affected by different environmental factors. RH was the main factor affecting most of the bulk soil modules (in six out of nine cases), but in addition, AT, SWP, soil pH, SOM and total N content were related to the changes in bacterial community structure of the bulk soil modules. In rhizosphere, the main measured environmental factor having an influence on the structure of different bacterial groups was AT, since five modules were related to this parameter but the relationships between RH, SWP, ST, and soil pH, and the structures of the modules were also revealed.

**Table 5:** **Statistically significant relationships (based on RDA analysis) between bulk and rhizosphere soil [whole community and pMENs bacterial community structure modules (marked with capital letters)], and air physical, bulk soil physic-chemical and short root morphological parameters**.

**Community/Module**	**Air and soil parameters**	**Variation explained %**	**Root morphology parameters**	**Variation explained %**
**BULK SOIL**				
Whole	(RH+AT)^***^	29.3	Dia^***^	15.6
A	(RH+pH)^***^	32.9	Dia^**^	16.5
B	(RH+SWP)^**^	39.6	Dia^**^	25.3
C	(RH+AT+SOM)^***^	37.0	Dia^*^	13.0
D	(pH+AT)^*^	22.7	TipW^*^	11.8
E			RTF_L_^*^	15.9
F	totN^*^	11.0	SRA^*^	12.5
G	(RH+SWP+pH)^***^	50.9	Dia^***^	24.0
H	RH^***^	29.0	Dia^***^	26.5
I	(RH+AT)^***^	29.3	TipW^*^	13.5
**RHIZOSPHERE SOIL**				
Whole	(RH+AT+SOM)^***^	40.6	Dia^***^	20.7
J	AT^***^	52.1	Dia^***^	45.0
K	(SWP+ST)^***^	43.6	TipW^**^	20.2
L	RH^***^	28.2	(Dia+RTF_L_)^***^	35.7
M	(pH+ST+AT)^***^	56.5		
N	AT^*^	21.8		
O	(AT+SWP)^***^	32.4	(Dia+TipL)^***^	31.3
P	(AT+RH)^***^	36.7	Dia^**^	22.7
Q	RH^***^	31.6	Dia^*^	12.3
R			(RTD+RTF_L_)^**^	24.2

*The means of the study years vegetation periods air relative humidity (RH), atmospheric temperature (AT), soil water potential (SWP), and soil temperature (ST) were used in the analyses. SOM, soil organic matter; totN, total nitrogen content; Dia, short root diameter; TipW, short root tip weight; TipL, root tip length; RTF_L_, root tip frequency per unit of length; SRA, short root area; RTD, root tissue density*.

* p < 0.05;

** p < 0.01;

*** *p < 0.001*.

Statistical analyses found a significant correlation between the ordination of experimental plots (first principal components) according to the bacterial community structure and leaf litter quality (u/b) for bulk soils: whole community (Spearman, *r* = −0.59 *p* < 0.05) and several pMEN modules (C *r* = −0.65 *p* < 0.01, G *r* = −0.69 *p* < 0.01, H *r* = −0.70 *p* < 0.01 and I *r* = −0.59 *p* < 0.05) as well as for rhizospheres: whole community (*r* = −0.65 *p* < 0.01) and pMEN modules (J *r* = −0.79 *p* < 0.001, K *r* = 0.50 *p* < 0.05, L *r* = 0.63 *p* < 0.05, N *r* = −0.57 *p* < 0.05, O *r* = 0.59 *p* < 0.05 and P *r* = −0.65 *p* < 0.01).

In addition, several environmental factors (air and soil physico-chemical and leaf litter quality parameters) affecting denitrifying bacterial community structure in the studied birch stand soils were found, but again the effect varied between bulk soil and rhizosphere (Table 6). In bulk soil, the analyses detected significant relationships only for *nirS%, nirS/nirK*, and *nir/nosZ*, while in rhizosphere, correlations for all the used denitrification gene parameters were obtained. RH was similarly related to *nirS%* and *nirS/nirK* in both bulk soil and rhizosphere while in the rhizosphere the relationship with *nosZII%* was also revealed. RH was related also to *nir/nosZ* in bulk soils and rhizospheres but the effect was opposite in these two soil compartments. SWP was strongly related to *nirS/nirK* in both soil compartments and in addition to *nirS%* in bulk soils and *nir/nosZ* in rhizospheres. AT had an almost similar relationship pattern to the RH in both soil compartments, but the effect was opposite to the RH. ST significantly affected *nirS/nirK* and *nir/nosZ* in both soil fractions, but in the case of *nir/nosZ* the effect was opposite to the bulk soil in rhizosphere, and more significant relationships between ST and gene parameters were revealed in rhizosphere. In addition, the *nirK* and *nir%* in rhizosphere bacterial community were correlated to soil pH. The statistical analyses detected relationships between u/b and denitrification gene proportions in both soil fractions, but the effect was not similar.

**Table 6:** **Statistically significant Spearman correlations between the studied gene parameters and environmental and root morphology parameters in studied bulk soil and rhizosphere samples**.

**SC**	**Gene parameters**	**Air parameters**		**Soil parameters**			**Root morphological parameters**							**LQ**
		**RH**	**AT**	**SWP**	**ST**	**pH**	**RTD**	**Dia**	**TipW**	**TipL**	**SRL**	**RTF_L_**	**RTF_W_**	**u/b**
Bulk soil	*nirS*%	0.61^*^	−0.61^*^	0.59^*^				−0.51^*^			0.54^*^	−0.56^*^		0.68^**^
	*nirS/nirK*	0.70^**^	−0.70^**^	0.74^***^	−0.56^*^			−0.61^*^	−0.56^*^		0.65*^*^		0.56^*^	0.72^**^
	*nir/nosZ*	0.56^*^	−0.60^*^		−0.52^*^				−0.61^*^				0.58^*^	0.64^**^
Rhizosphere	*nirS%*	0.50^*^	−0.55^*^		−0.57^*^			−0.69^**^			0.58^*^			
	*nirK%*					−0.67*^*^	0.63^*^							
	*nir%*					−0.72^**^	0.69^*^							
	*nirS/nirK*	0.88^***^	−0.88^***^	0.69^**^	−0.53^*^			−0.78^***^	−0.55^*^	0.54^*^	0.88^***^	−0.63^**^	0.53^*^	0.61^*^
	*nosZI%*				−0.73^*^		0.55^*^	−0.53^*^	−0.51^*^					0.73^**^
	*nosZII%*	0.63^**^	−0.65^**^		−0.72^*^			−0.80^***^			0.71^**^			0.61^*^
	*nosZI/nosZII*							−0.64^**^			0.54^*^			
	*nosZ%*		−0.51^*^		−0.78^***^		0.51^*^	−0.60^*^	−0.56^*^		0.56^*^		0.53^*^	0.73^**^
	*nir/nosZ*	−0.61^*^	0.64^**^	−0.63^**^	0.70^**^		−	0.54^*^	0.62^*^		−0.66^**^		−0.58^*^	−0.78^***^

* p < 0.05;

** p < 0.01;

*** *p < 0.001*.

*SC, soil compartment; RH, relative air humidity; AT, air temperature; SWP, soil water potential; ST, soil temperature; LQ, leaf litter quality; u/b, ratio between understory and birch leaf litter mass; Dia, short root diameter; TipW, root tip weight; TipL, root tip length; SRL, specific root length; RTF_L_, root tip frequency per unit of length; RTF_W_, root tip frequency per unit of weight; RTD, root tissue density*.

### Relationships between soil bacterial community structure and birch absorptive root morphological parameters

The statistical analyses revealed relationships between bacterial 16S rRNA gene abundance in bulk soil and birch absorptive root parameters such as SRL, TipW and RTF_L_ (Spearman correlations *r* = −0.53, *p* < 0.05; *r* = 0.51, *p* < 0.05; and *r* = 0.51, *p* < 0.05, respectively). Among root morphological parameters, root diameter was most related to the bacterial community structure at the whole community and pMEN module levels in both bulk soils and rhizospheres (Table 5). In addition, some significant correlations were also found between pMEN module structure and TipW, RTF_L_, TipL, SRA, and RTD.

Morphological parameters were also related to the denitrifying bacterial community abundance in the studied soils (Table 6). Similarly to the environmental parameters, we obtained significant relationships only for *nirS*%, *nirS/nirK*, and *nir/nosZ* in bulk soils, while in the rhizosphere the correlations for all denitrification related gene parameters were obtained. The revealed relationships between Dia and gene parameters were in almost all cases negative (except for *nir/nosZ* in rhizosphere). SRL was related to the abundance of the different *nir* genes (*nirS%* and *nirS/nirK*) in bulk soil and rhizosphere bacterial communities, but in rhizospheres, SRL was related to most of the *nosZ* gene parameters as well as to *nir/nosZ. NirS/nirK* had the highest number of significant relationships with the root morphology in both bulk soil and rhizosphere, but *nosZ%* and *nir/nosZ* were also significantly related to the birch root morphology in rhizosphere.

## Discussion

### The effect of elevated air humidity on the soil bacterial community structure

The results of this study indicate changes in the soil bacterial community structure in response to the increased air humidity. Albeit bacterial community structure was during 6 months not impacted by reduced precipitation and soil water content (2–8%) in beach and conifer forest ecosystems across Germany (Felsmann et al., 2015), the structural shifts were revealed in both bulk soil and rhizosphere of silver birch in response to misting. Furthermore, we found that structural adaptations of the bacterial communities in response to the elevated humidity differ remarkably between bulk soil and birch rhizosphere. The network analysis revealed changes within bacterial community that were not detectable at the whole community analyses level.

The increase in air relative humidity and decrease in air temperature at humidified plots changed bacterial community structure in both soil compartments; however, the effect on the individual phylotypes and the consortia of phylotypes revealed by network analysis was not similar. The impact of humidification on these community fractions depended on the weather of the year. The chemical and physical conditions of the environment had stronger effect on bacterial community structure than tree root morphology; whereas the root effect was stronger in the case of rhizosphere. Humidification affected a number of phylotypes from various bacterial genera. For example, the proportion of bacteria belonging to the phylum *Verrucomicrobia* was increased in the rhizosphere microbial community of the humidified plots, where a slight water potential increase was also notable. Buckley and Schmidt (2001) have shown that the variation in the abundance of this bacterial group in soil was significantly explained by soil moisture conditions.

The effect of increased air humidity on different soil bacteria can be variable. Soil pH was previously shown to be one of the main factors shaping bacterial community structure (Preem et al., 2012; Bergkemper et al., 2016b; Sun et al., 2016) and affecting microbial activity in soil (Truu et al., 2008; Bergkemper et al., 2016a). Albeit the soil pH was significantly increased in the humidified plots, the effect of this factor on bacterial community structure was detectable only in the cases of few consortia (network modules). We found that the proportion of bacteria from phylum *Acidobacteria* was significantly decreased in the bulk soil of humidified plots. Several studies have shown that these bacteria were sensitive to pH elevation in soil (Kim et al., 2003; Jones et al., 2009; Chu et al., 2010).

The increased air humidity can alter the other organism groups in soil that are closely linked to the specific bacterial groups. The significant increase in *Ca. Xiphinematobacter* proportions in rhizosphere of humidified plots may refer to the increased number of plant parasitic nematodes in this soil compartment due to the changed environment. Bacteria belonging to this genus have previously been described as endosymbionts of the nematodes attacking rootlets of the plants (Koivisto and Braig, 2003; Atibalentja and Noel, 2008).

In addition to the increased air humidity effect, several significant differences in the bulk soil and rhizosphere bacterial community structure between the two study years can be attributed to the year-specific weather conditions and also to the ecosystem turn from agricultural to the forest ecosystem. Several authors have reported on the significant effect of land use changes on bacterial community structure (Francioli et al., 2014; Quiza et al., 2014; Gómez-Acata et al., 2014), accompanied by the shifts in its functional properties: carbon turnover (Kirschbaum et al., 2013; Quiza et al., 2014) and nitrogen cycling (Kirschbaum et al., 2013; Francioli et al., 2014; Mirza et al., 2014). Our results indicate that the change in leaf litter quality, due to the birch canopy expansion, and concordant understory leaf mass decrease during birch stand development, significantly affected denitrifying bacterial community composition and its potential to emit N_2_O into the environment in both soil fractions.

### Effect of elevated air humidity on soil bacterial community functioning

#### The changes in soil microbial community potential functions

Deviations in bacterial community functioning in response to the increased air humidity and concordant changes in soil organic matter quality can be concluded from the observed changes in community structure. The phylotypes which were affected by such environmental change were distributed across the whole communities (involved and not involved in networks) of the studied soil compartments. The gas measurements in the experimental plots showed a reduction in CO_2_ emission and CH_4_ consumption by the soil at elevated air humidity conditions (Hansen et al., 2013). Kukumägi et al. (2014) recorded significantly higher basal respiration activity (O_2_ consumption) in the bulk soils after the two seasons of misting. Our results indicate qualitative changes in the soil carbon turnover processes. *Acidobacteria* communities, whose proportions were decreased in soils of humidified plots, were shown to be central to carbon cycling and play a significant role in degradation of accumulated biomass in boreal ecosystems (Rawat et al., 2012). The proportions of some anaerobic organisms (such as *Geobacter* spp. in both soil fractions, *Clostridium* spp. in rhizosphere) increased in humidified plots, and point to the elevated potential of anaerobic degradation of homocyclic aromatic compounds (the second most abundant natural organic molecules; Boll et al., 2014) in these soils. In addition, the proportion of genus *Opitutus* showed an increase in rhizosphere of humidified plots. The members of this genus are strict anaerobes fermenting mono-, di- and polysaccharides and living in a syntrophic relationship with hydrogenotrophic methanogenic archaea (Chin and Janssen, 2002). The increase in abundance of *Gordonia* spp. refers to increased aerobic oxidative carbohydrate metabolism in bulk soils of humidified plots (Kim et al., 2000). These organisms can degrade or modify a wide range of natural and artificial compounds (including toxic environmental pollutants) that are not readily biodegradable (Arenskötter et al., 2004). Furthermore, aerobic cellulolytic rhizobacteria from genus *Cytophaga*, also known as organisms protecting plant roots from nematodes (Aballay et al., 2012), were also among the bacteria favoring conditions initiated by increased air humidity.

The proportions of some bacterial groups, actively participating in aerobic soil organic matter turnover, decreased in soil of humidified plots. For example, the proportions of all the ten known species from the genus *Streptacidophilus* that have a major role in organic matter turnover (possess chitinases and diastases) in acidic soils (Komaki et al., 2015) decreased in rhizospheres. The proportions of *Rhodanobacter* spp. (degrade highly chlorinated aliphatic compounds; Nalin et al., 1999) and *Microbacterium* spp. [capable of lignin (Taylor et al., 2012) and cellulose (Yeasmin et al., 2015) degradation] decreased in both soil fractions.

In forest ecosystems, nutrients driven from organic matter degradation and from mineral-weathering processes are closely linked in the nutrient cycle, where the mineral weathering can be accelerated or initiated by microorganisms. The proportions of several bacterial genera (i.e., *Geobacter, Pedomicrobium*, and *Clostridium*) known to participate in mineral (iron and/or manganese) oxido-reduction processes (Braun et al., 2009; Uroz et al., 2011; Boll et al., 2014), increased in response to elevated air humidity in soil. The relative abundances of *Solibacillus spp*. and the acidophilic heterotrophic genus *Acidobacterium* with dissimilatory iron reduction ability (Coupland and Johnson, 2008; Ding et al., 2015) decreased. The abundance of bacterial genera (*Rhodanobacter* and *Pseudomonas*) that harbor species possessing the ability to undertake phosphate solubilisation (Uroz et al., 2011) also decreased at elevated air humidity conditions. All these phylotypes (except *Acidobacterium*) were involved in two largest network modules (C and G) of bulk soil. In addition, members of the genus *Dysgonomonas* were negatively affected by humidification in the bulk soil fraction. This is related to the change in nitrogen-fixation by the soil bacterial community since several members of this genus are shown to be nitrogenase possessing diazotrophs (Inoue et al., 2015).

#### Changes in soil bacterial community potential to produce and reduce nitrous oxide

We evaluated the impact of elevated air humidity on the potential of soil bacterial community to produce greenhouse gas N_2_O via denitrification process. Our results indicate a significant difference between bulk soil and rhizosphere in the denitrifying bacterial community characteristics as well as in the responses to changes in environmental conditions. The results show that air humidity and soil water potential affected the structure of the denitrifying bacterial community (especially *nirS/nirK* ratio) in both soil fractions. Data analysis showed that the abundance of *nirS*-type denitrifiers in bacterial community and in particular in several network modules of both soil compartments as well as the abundance of *nirK*-type denitrifiers in the rhizosphere community was related to the humidification effect. Increased air humidity changed soil conditions (especially increased pH) that were unfavorable for *nirK* gene possessing bacteria in birch rhizosphere. This led to the decrease in the potential of the bacterial community to produce N_2_O via this process in this soil compartment. Similar community-dependent responses of denitrifiers to the changes in soil pH have also been reported in different wetlands (Dörsch et al., 2012; Ligi et al., 2014). Our results also indicate a decrease in organisms possessing *nosZ* genes (at the expense of *nosZ* clade I) in elevated air humidity conditions in the rhizosphere bacterial community. These results indicate that a substantial proportion of *nirK*-type denitrifiers possess *nosZ* clade I gene in birch stand rhizospheres. Although not revealed in this study, several other studies have shown similarities in relationships between environmental factors and *nirK* as well as *nosZ* clade I possessing organisms (Kandeler et al., 2006; Ligi et al., 2014). As indicated by the ratio between *nir* and *nosZ* genes, the potential for N_2_O production and concordant emission from soil was much bigger in the case of bulk soil bacteria if compared to the birch rhizosphere bacterial community. The increased air humidity did not have significant effects on this bacterial community in either soil compartment.

Hansen et al. (2013) reported low and sometimes even negative N_2_O fluxes (122 to 139 μg m^−2^ h^−1^ and −5 to 32 μg m^−2^ h^−1^ from the control and humidified plots, respectively) within the period of 2009 to 2010 from the same plots whereas significantly higher fluxes were measured from the control plots in a dryer year.

### Adaptation mechanisms of the soil bacterial-birch root system in response to the elevated air humidity

The results show that a change in bacterial community structure is linked to the morphology of birch ectomycorrhizal absorptive roots in soil of birch stand. This indicates a complex adaptation mechanism in the system, where the driving factor can be both a change in soil microbial community as well as a shift in plant physiology and morphology.

Bacterial network modules as well as denitrifying bacterial community parameters were mainly related to absorptive root diameter. In addition, the denitrifying bacterial community characteristics were related to the specific root length, which variation is in most cases determined by the root diameter (Ostonen et al., 2013; Parts et al., 2013). The increased length per mass unit of absorptive roots increases significantly the contact surface between roots and soil. It also increases the number of soil particles affected by roots thus creating space for rhizosphere bacteria. Thinner absorptive roots and increased SRL have been associated with decreasing nutrient availability (Ostonen et al., 2007; Zobel et al., 2007). The change in soil conditions of humidified plots was mediated by plant responses such as reduced transpiration flux (Kupper et al., 2011), changed hydraulic architecture (Sellin et al., 2013) and higher fine root biomass in trees (Rosenvald et al., 2014). The results of this study indicate that shifts in root morphology have a significant effect on the microbial community in the rhizosphere. Previous studies in the FAHM experiment have shown that humidification substantially increased (by 20%) the proportion of ectomycorrhizal (EcM) absorptive roots in the total fine root mass (Rosenvald et al., 2014) An almost 25% increase in SRL of EcM absorptive roots after the 2 years of humidification coincided with a shift in EcM fungal colonizers toward the dominance of hydrophilic taxa (Parts et al., 2013).

The results also show a positive relationship between root branching intensity (RTFw) and denitrification-related gene parameters (*nirS/nirK* and *nir/nosZ*) in bulk soil with respect to the effect of active root tips on denitrifying bacterial community structure in the soil compartment. While microbial activity is limited, especially by organic carbon availability, the essential increase in root surface area or root branching intensity may lead to higher input of labile root exudates in the rhizosphere (Cesco et al., 2012). Furthermore, a positive correlation between *nirS/nirK* in bulk soil and rhizosphere as well as *nosZII/nosZI* in rhizosphere and SRL of ectomycorrhizal root tips indicate favorable conditions for *nirS* and *nosZ* clade II genes possessing bacteria in soil of silver birch stand at increased air humidity conditions.

The structural change in bacterial community triggered by elevated air humidity may induce the changes in root morphology and essentially affect root foraging, as shown for EcM fungi. Modifications induced by bacteria (*Burkholderia glathei*) in pine root morphology (increased number of lateral roots and root hairs) and enhanced mineral nutrition of trees via increased mineral weathering have been demonstrated under experimental conditions (Calvaruso et al., 2006).

## Conclusions

The increased air humidity induced alteration in soil bacterial community structure in silver birch stands. The bulk soil and rhizosphere bacterial communities were different and responded differently to humidification treatment. The changes in bacterial community structure were related to the modified soil abiotic and biotic variables and were also dependent on the weather conditions of the year. Increased soil pH affected the abundance of *Acidobacteria*, some co-occurring phylotypes in bulk soil bacterial community and abundance of *nirK* gene possessing denitrifying bacterial fraction in birch rhizosphere. As a result of elevated air humidity on bacterial community structure, changes in carbon metabolism and concordant shifts in soil organic carbon quality can be assumed. Alterations in many other crucial microbial functions such as phosphorus and nitrogen turnover as well as in mineral weathering in deciduous forest soils in response to increased air humidity in the Baltic region can be forecasted from the results of this study. The short term air humidity elevation decreased the denitrification potential of the rhizosphere bacterial community, but the emission potential of the N_2_O via denitrification was not affected in silver birch stand soil. The results of this humidity manipulation study reveal that the shifts in soil bacterial community structure are strongly linked to the changed tree absorptive root morphology and physiology.

## Author contributions

MT, IO, JP, KL, TL, HN, KR, KP, and PK performed the experiments. MT, JT, and IO wrote the manuscript. MT, IO, KL, and JT designed and supervised the study.

## Funding

This study was supported by the Estonian Ministry of Education and Research (Grant IUT2-16 and IUT34-9), the Estonian Science Foundation (Grant 7792) and by the European Regional Development Fund through the Centre of Excellence EcolChange at the University of Tartu.

### Conflict of interest statement

The authors declare that the research was conducted in the absence of any commercial or financial relationships that could be construed as a potential conflict of interest.
