# Supplementary material for: Elevated Air Humidity Changes Soil Bacterial Community Structure in the Silver Birch Stand
Source: Front Microbiol. 2017 Apr 3;8:557. doi: 10.3389/fmicb.2017.00557 (PMC5376589; doi:10.3389/fmicb.2017.00557)
Supplement: Supplementary file 1 [file DataSheet1.docx]

Supplementary Material

Soil bacterial community structure changes in response to the elevated air humidity in silver birch stand

Marika Truu^1*^, Ivika Ostonen^1^, Jens-Konrad Preem^1^, Krista Lõhmus^2^, Teele Ligi^1^, Hiie Nõlvak^1^, Katrin Rosenvald^2^, Priit Kupper^2^, Jaak Truu^1^

*** Correspondence:** Marika Truu: marika.truu@ut.ee

# Supplementary Figures and Tables

**Supplementary Table 1.** The meteorological conditions (annual (A) mean air temperatures (AT; °C) and sums of annual and vegetation periods (Veg. per.) precipitation(P; mm) at the study site; means of the vegetation periods relative air humidity (RH; %) and air temperatures, and means of annual and vegetation periods soil physical parameters values (temperature (ST; °C) and water potential (SWP; kPA)) at the experimental plots (control (C; n=4) and humidified (H; n=4)) in 15 cm of soil during the humidification experiment.

|  | 2008* | | | | 2009 | | | | 2010 | | | | 2011** | | | |
| --- | --- | --- | --- | --- | --- | --- | --- | --- | --- | --- | --- | --- | --- | --- | --- | --- |
|  | A | | Veg. per. | | A | | Veg. per. | | A | | Veg. per. | | A | | Veg. per. | |
| Plots | C | H | C | H | C | H | C | H | C | H | C | H | C | H | C | H |
| P | 934 | | 455 | | 827 | | 378 | | 873 | | 407 | | 541 | | 195 | |
| AT | 5.7 | | 13.5 | 13.7 | 5.1 | | 12.4 | 12.0 | 5.0 | | 13.7 | 13.6 | 7.0 | | 14.2 | 14.1 |
| SWP | -52.7 | -12.4 | -70.2 | -16.4 | -41.3 | -3.8 | -82.5 | -7.5 | -30.5 | -25.8 | -60.8 | -51.5 | -78.8 | -75.1 | -131.3 | -124.5 |
| ST | 10.2 | 10.3 | 12.4 | 12.6 | 6.8 | 7.0 | 12.0 | 12.3 | 7.5 | 7.6 | 13.1 | 13.2 | 8.6 | 8.5 | 13.1 | 13.2 |
| RH | nd | nd | 79.8 | 81.5 | nd | nd | 81.3 | 82.5 | nd | nd | 80.1 | 81.6 | nd | nd | 78.7 | 80.4 |

*Annual mean parameter values for plots are calculated using May to December data; **Annual mean parameter values for plots are calculated using January to October data; nd – the parameter was not detected during nonvegetation period.

**Supplementary Table 2.** Characteristics of qPCR primer sets used in this study.

| Primer | Sequence 5’-3’ | Target  gene | Amplicon size (bp) | Primer concentration (µM) | Annealing temperature (°C) | Reference |
| --- | --- | --- | --- | --- | --- | --- |
| L-V6 | CAACGCGARGAACCTTACC | 16S rRNA | 112 | 0.4 | 54 | Gloor et al. (2010) |
| R-V6 | ACAACACGAGCTGACGAC |  |  | 0.4 |  |  |
| nirSCd3aF | AACGYSAAGGARACSGG | *nirS* | 425 | 0.8 | 51 | Kandeler et al. (2006) |
| nirSR3d | GASTTCGGRTGSGTCTTSAYGAA |  |  | 0.8 |  |  |
| FlaCu | ATCATGGTSCTGCCGCG | *nirK* | 473 | 0.8 | 55 | Hallin and Lindgren (1999) |
| R3Cu | GCCTCGATCAGRTTGTGGTT |  |  | 0.8 |  |  |
| nosZF | CGYTGTTCMTCGACAGCCAG | *nosZ* I | 453 | 0.9 | 60 | Kloos et al. (2001) |
| nosZ1622R | CGSACCTTSTTGCCSTYGCG |  |  | 0.9 |  | Throbäck et al. (2004) |
| nosZIIF | CTIGGICCIYTKCAYAC | *nosZ* II | ~700 | 0.9 | 52 | Jones et al. (2013) |
| nosZIIR | GCIGARCARAAITCBGTRC |  |  | 0.9 |  |  |

**Supplementary Table 3.** Mean and standard deviations of the bacterial 16S sRNA gene copy numbers and proportions of the total *nir and nosZ* genes, and the ratios of *nirS* and *nirK* genes, *nosZI* and *nosZII* genes and total *nir* and *nosZ* gens in the studied soil compartments (Soil comp.) of the control (n=4) and humidified (Humid., n=4) plots at both study years. Nd, not detected.

| **Soil comp.** | **Treat.** | **16S rRNA**  **(x10^9^copies g^-1^dw^-1^)** | | ***nir***  **(%)** | | ***nirS/nirK*** | | ***nosZ***  **(%)** | | ***nosZ II/nosZ I*** | | ***nir/nosZ*** | |
| --- | --- | --- | --- | --- | --- | --- | --- | --- | --- | --- | --- | --- | --- |
|  |  | **2009** | **2011** | **2009** | **2011** | **2009** | **2011** | **2009** | **2011** | **2009** | **2011** | **2009** | **2011** |
| **Bulk**  **soil** | **Control** | 4.55±2.04 | 6.12±1.28 | 10.72±2.99 | 9.57±1.48 | 0.76±0.26 | 0.52±0.08 | 0.89±0.32 | 0.94±0.21 | 0.62±0.29 | 0.56±0.20 | 12.3±1.3 | 10.3±1.8 |
|  | **Humid.** | 3.84±0.84 | 8.01±0.69 | 11.13±2.35 | 9.30±0.82 | 0.74±0.07 | 0.59±0.11 | 0.84±0.14 | 0.92±0.15 | 0.46±0.20 | 0.54±0.13 | 13.4±2.8 | 10.3±1.7 |
| **Rhizo-**  **sphere** | **Control** | nd | nd | 16.70±2.00 | 12.19±1.29 | 0.33±0.04 | 0.22±0.03 | 5.38±0.98 | 2.92±0.65 | 0.13±0.05 | 0.07±0.05 | 3.14±0.28 | 4.39±1.31 |
|  | **Humid.** | nd | nd | 10.85±5.06 | 10.73±1.30 | 0.57±0.16 | 0.27±0.06 | 3.81±1.27 | 2.13±0.23 | 0.13±0.07 | 0.08±0.03 | 2.86±0.93 | 5.12±1.02 |

**Supplementary Table 4.** Topological properties of the empirical phylogenetic molecular ecological networks of microbial communities and their associated random phylogenetic molecular ecological networks for bulk soil and rhizosphere samples. Shown are number of modules used in further analyses (with at least five OTUs), the number of all obtained modules is given in in the brackets. AC, average connectivity.

| Soil compartment | No. of original OTUs | Nodes | Edges | AC | Average geodesic distance^a^ | Average clustering coefficient^a^ | Modularity | Number of modules | R^2^ of powerlaw |
| --- | --- | --- | --- | --- | --- | --- | --- | --- | --- |
| Bulk soil | 1110 | 229 | 501 | 4.38 | 3.343 ±0.089 | 0.039 ± 0.008 | 0.52 | 11 | 0.886 |
| Rhizosphere | 1110 | 210 | 326 | 3.11 | 4.109 ± 0.133 | 0.022 ± 0.009 | 0.67 | 16 | 0.950 |

^a^Calculated by 100 times randomly rewiring all of the links of a phylogenetic molecular ecological network.

Network’s topological properties acquired from 100 randomized phylogenetic networks

| Soil | Average geodesic distance*^a^* | Average clustering coefficient^a^ | Modularity |
| --- | --- | --- | --- |
| Bulk Soil | 3.176± 0.076 | 0.060± 0.007 | 0.347± 0.006 |
| Rhizosphere | 3.317± 0.103 | 0.042± 0.007 | 0.406± 0.006 |

^a^Calculated by 100 times randomly rewiring all of the links of the phylogenetic molecular ecological networks.

**Supplementary Table 5.** Phylogenetic affiliation of the phylotypes (OTUs) of bulk soil and rhizosphere pMENs modules (Mod) and OTUs that were not involved in pMENs but that proportions were significantly different (LDA score >2) between control and humidified plots according to the LefSe analyses. The modules affected by humidification and these phylotypes that differed by their abundances (p<0.05) in control and humidified plots soils are marked with asterisks. *Ca.*, *Candidatus*

| Mod. | | OTU | Phylum | Class | Order | | Family | Genera |
| --- | --- | --- | --- | --- | --- | --- | --- | --- |
| BULK SOIL | | | | | | | | |
| A | | 25 | *Bacteroidetes* | *Sphingobacteria* | *Sphingobacteriales* | | unclassified | unclassified |
|  |  | 84 | *Verrucomicrobia* | *Spartobacteria* | *Spartobacteriales* | | *Spartobacteriaceae* | *Ca. Xiphinematobacter* |
|  |  | 124 | *Planctomycetes* | unclassified | unclassified | | unclassified | unclassified |
|  |  | 138 | *Planctomycetes* | unclassified | unclassified | | unclassified | unclassified |
|  |  | 155 | *Acidobacteria* | Sva0725 | Sva0725 | | unclassified | unclassified |
|  |  | 211 | *Planctomycetes* | vadinHA49 | unclassified | | unclassified | unclassified |
|  |  | 282 | *Chloroflexi* | Bljii12 | B07_WMSP1 | | FFCH4570 | unclassified |
|  |  | 289 | *Planctomycetes* | PW285 | unclassified | | unclassified | unclassified |
|  |  | 611 | *Actinobacteria* | *Actinobacteria* | *Actinomycetales* | | unclassified | unclassified |
|  |  | 674 | *Proteobacteria* | *Betaproteobacteria* | unclassified | | unclassified | unclassified |
|  |  | 734 | *Acidobacteria* | *Acidobacteria* | *Acidobacteriales* | | *Acidobacteriaceae* | unclassified |
|  |  | 765 | *Proteobacteria* | *Deltaproteobacteria* | *Syntrophobacterales* | | *Syntrophobacteraceae* | unclassified |
| B* | | 11* | *Actinobacteria* | *Actinobacteria* | *Actinomycetales* | | *Microbacteriaceae* | *Microbacterium* |
|  |  | 41 | *Proteobacteria* | *Betaproteobacteria* | *Burkholderiales* | | *Comamonadaceae* | unclassified |
|  |  | 62 | *Verrucomicrobia* | *Opitutae* | *Opitutales* | | *Opitutaceae* | *Opitutus* |
|  |  | 112* | *WPS-2* | unclassified | unclassified | | unclassified | unclassified |
|  |  | 143 | *ZB2* | unclassified | unclassified | | unclassified | unclassified |
|  |  | 149 | *Proteobacteria* | unclassified | unclassified | | unclassified | unclassified |
|  |  | 157* | *TM7* | unclassified | unclassified | | unclassified | unclassified |
|  |  | 159 | *Firmicutes* | *Bacilli* | *Bacillales* | | *Bacillaceae* | *Bacillus* |
|  |  | 179 | *Actinobacteria* | *Actinobacteria* | *Actinomycetales* | | *Streptomycetaceae* | *Streptomyces* |
|  |  | 215 | *Bacteroidetes* | unclassified | unclassified | | unclassified | unclassified |
|  |  | 233 | *Bacteroidetes* | *Flavobacteria* | *Flavobacteriales* | | *Flavobacteriaceae* | unclassified |
|  |  | 371 | *Proteobacteria* | unclassified | unclassified | | unclassified | unclassified |
|  |  | 455* | *Proteobacteria* | *Gammaproteobacteria* | *Enterobacteriales* | | *Enterobacteriaceae* | unclassified |
|  |  | 456* | *Proteobacteria* | *Gammaproteobacteria* | *Chromatiales* | | unclassified | unclassified |
|  |  | 478 | *Verrucomicrobia* | *Verrucomicrobiae* | *Verrucomicrobiales* | | *Verrucomicrobia* sd. 3 | unclassified |
|  |  | 556 | *Chloroflexi* | *Anaerolineae* | *Caldilineales* | | *Caldilineaceae* | *Caldilinea* |
|  |  | 678 | *Proteobacteria* | *Gammaproteobacteria* | unclassified | | unclassified | unclassified |
|  |  | 683 | *Proteobacteria* | *Betaproteobacteria* | *Burkholderiales* | | unclassified | unclassified |
| C | | 19 | *Proteobacteria* | *Betaproteobacteria* | *Rhodocyclales* | | unclassified | unclassified |
|  |  | 21 | *Planctomycetes* | *Phycisphaerae* | unclassified | | unclassified | unclassified |
|  |  | 32 | *Acidobacteria* | *Chloracidobacteria* | unclassified | | unclassified | unclassified |
|  |  | 33 | *Proteobacteria* | *Alphaproteobacteria* | *Rhodospirillales* | | *Acetobacteraceae* | unclassified |
|  |  | 49 | *Proteobacteria* | *Deltaproteobacteria* | *Desulfuromonadales* | | *Geobacteraceae* | *Geobacter* |
|  |  | 71 | SPAM | 0319-6G9 | unclassified | | unclassified | unclassified |
|  |  | 78 | *Chloroflexi* | *Anaerolineae* | A31 | | S47 | unclassified |
|  |  | 90 | *Firmicutes* | *Bacilli* | *Bacillales* | | unclassified | unclassified |
|  |  | 140 | *Actinobacteria* | *Actinobacteria* | *Actinomycetales* | | *Nocardioidaceae* | *Kribbella* |
|  |  | 164 | *Proteobacteria* | unclassified | unclassified | | unclassified | unclassified |
|  |  | 173 | *Planctomycetes* | agg27 | OM190 | | unclassified | unclassified |
|  |  | 184 | *Proteobacteria* | *Gammaproteobacteria* | *Legionellales* | | *Legionellaceae* | *Legionella* |
|  |  | 193 | *Actinobacteria* | *Actinobacteria* | 0319-7L14 | | unclassified | unclassified |
|  |  | 196 | *Proteobacteria* | *Deltaproteobacteria* | MIZ46 | | unclassified | unclassified |
|  |  | 204 | *Chloroflexi* | SOGA31 | unclassified | | unclassified | unclassified |
|  |  | 209 | *Planctomycetes* | FFCH393 | unclassified | | unclassified | unclassified |
|  |  | 223 | *Bacteroidetes* | *Sphingobacteria* | *Sphingobacteriales* | | *Flexibacteraceae* | *Dyadobacter* |
|  |  | 235 | *Bacteroidetes* | unclassified | unclassified | | unclassified | unclassified |
|  |  | 241 | *Actinobacteria* | *Actinobacteria* | *Solirubrobacterales* | | *Patulibacteraceae* | unclassified |
|  |  | 257 | *Firmicutes* | *Clostridia* | *Clostridiales* | | *Clostridiaceae* | *Caloramator* |
|  |  | 275 | TM7 | TM7-1 | unclassified | | unclassified | unclassified |
|  |  | 296 | *Firmicutes* | *Clostridia* | *Clostridiales* | | *Peptococcaceae* | *Desulfosporosinus* |
|  |  | 328 | *Acidobacteria* | PAUC37f | unclassified | | unclassified | unclassified |
|  |  | 329 | *Proteobacteria* | Deltaproteobacteria | *Desulfuromonadales* | | *Geobacteraceae* | *Geobacter* |
|  |  | 332 | *Chloroflexi* | Bljii12 | AKYG885 | | 5B-12 | unclassified |
|  |  | 347 | *Proteobacteria* | *Alphaproteobacteria* | *Rhodospirillales* | | *Rhodospirillaceae* | unclassified |
|  |  | 368 | *Proteobacteria* | *Alphaproteobacteria* | *Rhizobiales* | | *Hyphomicrobiaceae* | *Pedomicrobium* |
|  |  | 378 | *Actinobacteria* | *Actinobacteria* | *Actinomycetales* | | unclassified | unclassified |
|  |  | 397 | *Proteobacteria* | *Gammaproteobacteria* | *Legionellales* | | *Coxiellaceae* | *Aquicella* |
|  |  | 468 | *Acidobacteria* | *Chloracidobacteria* | unclassified | | unclassified | unclassified |
|  |  | 510 | *Proteobacteria* | *Gammaproteobacteria* | *Pseudomonadales* | | *Pseudomonadaceae* | *Pseudomonas* |
|  |  | 572 | *Actinobacteria* | *Actinobacteria* | *Acidimicrobiales* | | unclassified | unclassified |
|  |  | 578 | *Acidobacteria* | *Chloracidobacteria* | unclassified | | unclassified | unclassified |
|  |  | 595 | *Firmicutes* | *Bacilli* | *Bacillales* | | *Paenibacillaceae* | *Paenibacillus* |
|  |  | 607 | *Proteobacteria* | *Alphaproteobacteria* | *Rhodospirillales* | | *Acetobacteraceae* | unclassified |
|  |  | 646 | *Actinobacteria* | *Actinobacteria* | *Actinomycetales* | | unclassified | unclassified |
|  |  | 722 | *Actinobacteria* | *Actinobacteria* | *Actinomycetales* | | *Streptomycetaceae* | *Streptomyces* |
|  |  | 724 | *Proteobacteria* | *Alphaproteobacteria* | *Rhodobacterales* | | *Rhodobacteraceae* | *Amaricoccus* |
|  |  | 731 | *Proteobacteria* | *Deltaproteobacteria* | MIZ46 | | unclassified | unclassified |
|  |  | 754 | *Proteobacteria* | *Deltaproteobacteria* | *Desulfuromonadales* | | *Geobacteraceae* | *Geobacter* |
|  |  | 759 | *Acidobacteria* | *Chloracidobacteria* | unclassified | | unclassified | unclassified |
|  |  | 767 | *Acidobacteria* | *Acidobacteria-5* | unclassified | | unclassified | unclassified |
| D | | 5 | *Firmicutes* | *Clostridia* | *Clostridiales* | | unclassified | unclassified |
|  |  | 31 | *Acidobacteria* | *Solibacteres* | *Solibacterales* | | *Solibacteraceae* | *Ca. Solibacter* |
|  |  | 58 | *Acidobacteria* | *Solibacteres* | *Solibacterales* | | *Solibacteraceae* | *Ca. Solibacter* |
|  |  | 68 | *Bacteroidetes* | *Flavobacteria* | *Flavobacteriales* | | *Flavobacteriaceae* | *Flavobacterium* |
|  |  | 69 | *Acidobacteria* | *Solibacteres* | *Solibacterales* | | *Solibacteraceae* | *Ca. Solibacter* |
|  |  | 75 | *Acidobacteria* | *Chloracidobacteria* | unclassified | | unclassified | unclassified |
|  |  | 85 | *Proteobacteria* | *Alphaproteobacteria* | *Rhizobiales* | | *Hyphomicrobiaceae* | unclassified |
|  |  | 91 | WS3 | PRR-12 | Sediment-1 | | PRR-10 | unclassified |
|  |  | 170 | *Proteobacteria* | *Alphaproteobacteria* | *Rickettsiales* | | *Rickettsiaceae* | *Rickettsia* |
|  |  | 202 | *Proteobacteria* | *Deltaproteobacteria* | MIZ46 | | unclassified | unclassified |
|  |  | 251 | *Bacteroidetes* | *Sphingobacteria* | *Sphingobacteriales* | | *Sphingobacteriaceae* | *Pedobacter* |
|  |  | 286 | *Actinobacteria* | *Actinobacteria* | *Actinomycetales* | | unclassified | unclassified |
|  |  | 336 | *Actinobacteria* | *Actinobacteria* | *Actinomycetales* | | *Microbacteriaceae* | *Yonghaparkia* |
|  |  | 340 | *Proteobacteria* | unclassified | unclassified | | unclassified | unclassified |
|  |  | 351 | *Acidobacteria* | *Solibacteres* | *Solibacterales* | | *Solibacteraceae* | *Ca. Solibacter* |
|  |  | 388 | *Acidobacteria* | *Chloracidobacteria* | unclassified | | unclassified | unclassified |
|  |  | 407 | *Proteobacteria* | *Alphaproteobacteria* | unclassified | | unclassified | unclassified |
|  |  | 436 | TM7 | TM7-1 | unclassified | | unclassified | unclassified |
|  |  | 443 | *Planctomycetes* | agg27 | OM190 | | unclassified | unclassified |
|  |  | 444 | *Chloroflexi* | *Anaerolineae* | envOPS12 | | unclassified | unclassified |
|  |  | 450 | *Verrucomicrobia* | *Opitutae* | *Opitutales* | | *Opitutaceae* | unclassified |
|  |  | 467 | *Chloroflexi* | unclassified | unclassified | | unclassified | unclassified |
|  |  | 473 | *Proteobacteria* | *Alphaproteobacteria* | *Sphingomonadales* | | *Sphingomonadaceae* | *Novosphingobium* |
|  |  | 476 | *Actinobacteria* | *Actinobacteria* | unclassified | | unclassified | unclassified |
|  |  | 518 | *Chloroflexi* | Bljii12 | AKYG885 | | Dolo_23 | unclassified |
|  |  | 575 | *Verrucomicrobia* | *Verrucomicrobiae* | *Verrucomicrobiales* | | *Verrucomicrobia* sd. 3 | unclassified |
|  |  | 587 | *Proteobacteria* | *Deltaproteobacteria* | *Syntrophobacterales* | | *Syntrophobacteraceae* | unclassified |
|  |  | 603 | *Acidobacteria* | *Solibacteres* | *Solibacterales* | | *Solibacteraceae* | *Ca. Solibacter* |
|  |  | 612 | *Proteobacteria* | *Deltaproteobacteria* | *Bdellovibrionales* | | *Bdellovibrionaceae* | *Bdellovibrio* |
|  |  | 632 | *Actinobacteria* | *Actinobacteria* | *Actinomycetales* | | *Nocardioidaceae* | unclassified |
|  |  | 654 | *Chloroflexi* | *Anaerolineae* | SJA-15 | | unclassified | unclassified |
|  |  | 771 | *Actinobacteria* | *Actinobacteria* | unclassified | | unclassified | unclassified |
| E | | 44 | *TM7* | unclassified | unclassified | | unclassified | unclassified |
|  |  | 160 | *Firmicutes* | *Bacilli* | *Bacillales* | | *Paenibacillacea* | unclassified |
|  |  | 217 | *Planctomycetes* | *Planctomycea* | *Gemmatales* | | *Gemmataceae* | unclassified |
|  |  | 404 | *Actinobacteria* | *Actinobacteria* | *Actinomycetales* | | *Thermomonosporaceae* | unclassified |
|  |  | 544 | *Proteobacteria* | *Betaproteobacteria* | *Methylophilales* | | *Methylophilaceae* | unclassified |
|  |  | 659 | *Elusimicrobia* | *Elusimicrobia* | *FAC88* | | *unclassified* | unclassified |
| F | | 22 | *Actinobacteria* | *Actinobacteria* | *Actinomycetales* | | *Nakamurellaceae* | unclassified |
|  |  | 56 | *Chloroflexi* | *Chloroflexi* | *Roseiflexales* | | *Kouleothrixaceae* | unclassified |
|  |  | 67 | *Bacteroidetes* | *Sphingobacteria* | *Sphingobacteriales* | | unclassified | unclassified |
|  |  | 74 | *Firmicutes* | *Bacilli* | *Bacillales* | | *Bacillaceae* | *Bacillus* |
|  |  | 104 | *Bacteroidetes* | *Flavobacteria* | *Flavobacteriales* | | *Flavobacteriaceae* | *Chryseobacterium* |
|  |  | 133 | *Acidobacteria* | unclassified | unclassified | | unclassified | unclassified |
|  |  | 268 | *Verrucomicrobia* | unclassified | unclassified | | unclassified | unclassified |
|  |  | 283 | *AD3* | ABS-6 | unclassified | | unclassified | unclassified |
|  |  | 440 | *Acidobacteria* | *Solibacteres* | *Solibacterales* | | *Solibacteraceae* | *Ca. Solibacter* |
|  |  | 460 | *Actinobacteria* | *Actinobacteria* | MC47 | | unclassified | unclassified |
|  |  | 548 | *Planctomycetes* | C6 | unclassified | | unclassified | unclassified |
|  |  | 565 | *Proteobacteria* | unclassified | unclassified | | unclassified | unclassified |
|  |  | 640 | *Proteobacteria* | *Deltaproteobacteria* | *Desulfuromonadales* | | *Geobacteraceae* | *Geobacter* |
|  |  | 655 | *Verrucomicrobia* | *Verrucomicrobiae* | *Verrucomicrobiales* | | *Verrucomicrobiaceae* | *Prosthecobacter* |
|  |  | 663 | *Bacteroidetes* | *Sphingobacteria* | *Sphingobacteriales* | | *Sphingobacteriaceae* | *Pedobacter* |
|  |  | 738 | *Bacteroidetes* | *Sphingobacteria* | *Sphingobacteriales* | | unclassified | unclassified |
|  |  | 779 | *Proteobacteria* | *Gammaproteobacteria* | *Pseudomonadales* | | *Pseudomonadaceae* | *Pseudomonas* |
|  |  | 950 | *Proteobacteria* | *Alphaproteobacteria* | unclassified | | unclassified | unclassified |
| G* | | 3 | *Acidobacteria* | *Acidobacteria-5* | unclassified | | unclassified | unclassified |
|  |  | 26 | *Actinobacteria* | *Actinobacteria* | *Actinomycetales* | | *Nocardioidaceae* | *Marmoricola* |
|  |  | 45 | *Proteobacteria* | *Gammaproteobacteria* | *Enterobacteriales* | | *Enterobacteriaceae* | unclassified |
|  |  | 102 | *Firmicutes* | *Bacilli* | *Lactobacillales* | | *Streptococcaceae* | *Streptococcus* |
|  |  | 110 | *Proteobacteria* | unclassified | unclassified | | unclassified | unclassified |
|  |  | 121 | *Proteobacteria* | *Gammaproteobacteria* | unclassified | | unclassified | unclassified |
|  |  | 154 | *Gemmatimonadetes* | *Gemmatimonadetes* | *Gemmatimonadales* | | *Gemmatimonadaceae* | *Gemmatimonas* |
|  |  | 162 | *Proteobacteria* | *Betaproteobacteri* | *Burkholderiales* | | *Oxalobacteraceae* | *Herbaspirillum* |
|  |  | 212 | *Acidobacteria* | *Acidobacteria* | *Acidobacteriales* | | *Acidobacteriaceae* | unclassified |
|  |  | 259* | *Actinobacteria* | *Actinobacteria* | *Actinomycetales* | | *Mycobacteriaceae* | *Mycobacterium* |
|  |  | 294 | *Actinobacteria* | *Actinobacteria* | *Actinomycetales* | | *Catenulisporaceae* | *Catenulispora* |
|  |  | 312 | *Actinobacteria* | *Actinobacteria* | *Actinomycetales* | | *Micromonosporaceae* | *Micromonospora* |
|  |  | 326 | *Actinobacteria* | *Actinobacteria* | *Actinomycetales* | | *Microbacteriaceae* | *Microbacterium* |
|  |  | 327 | *Actinobacteria* | *Actinobacteria* | *Actinomycetales* | | *Actinospicaceae* | unclassified |
|  |  | 344 | *Actinobacteria* | *Actinobacteria* | *Actinomycetales* | | *Micrococcaceae* | *Arthrobacter* |
|  |  | 359 | *Proteobacteria* | *Betaproteobacteria* | *Burkholderiales* | | *Burkholderiaceae* | *Burkholderia* |
|  |  | 387 | *Actinobacteria* | *Actinobacteria* | *Acidimicrobiales* | | EB1017 | unclassified |
|  |  | 399* | *Proteobacteria* | *Gammaproteobacteria* | *Xanthomonadales* | | *Xanthomonadaceae* | *Rhodanobacter* |
|  |  | 462 | *Firmicutes* | *Bacilli* | *Bacillales* | | *Planococcaceae* | *Solibacillus* |
|  |  | 469 | *Actinobacteria* | *Actinobacteria* | *Actinomycetales* | | unclassified | unclassified |
|  |  | 502 | *Proteobacteria* | *Alphaproteobacteria* | *Rhodospirillales* | | *Acetobacteraceae* | unclassified |
|  |  | 505 | *Verrucomicrobia* | *Opitutae* | *Opitutales* | | *Opitutaceae* | *Opitutus* |
|  |  | 532 | *Actinobacteria* | *Actinobacteria* | *Actinomycetales* | | *Geodermatophilaceae* | *Geodermatophilus* |
|  |  | 533 | *Verrucomicrobia* | *Verrucomicrobiae* | *Verrucomicrobiales* | | *Verrucomicrobiaceae* | *Prosthecobacter* |
|  |  | 577 | *Proteobacteria* | *Gammaproteobacteria* | unclassified | | unclassified | unclassified |
|  |  | 619 | *Acidobacteria* | *Solibacteres* | *Solibacterales* | | *Solibacteraceae* | *Ca. Solibacter* |
|  |  | 642 | *Gemmatimonadetes* | *Gemmatimonadetes* | *Gemmatimonadales* | | *Gemmatimonadaceae* | *Gemmatimonas* |
|  |  | 647 | *WS3* | PRR-12 | Sediment-1 | | unclassified | unclassified |
|  |  | 648 | *Actinobacteria* | unclassified | unclassified | | unclassified | unclassified |
|  |  | 657 | *Proteobacteria* | *Gammaproteobacteria* | *Pseudomonadales* | | *Pseudomonadaceae* | *Pseudomonas* |
|  |  | 681 | *Acidobacteria* | *Acidobacteria* | *Acidobacteriales* | | *Acidobacteriaceae* | unclassified |
|  |  | 699 | *Bacteroidetes* | *Sphingobacteria* | *Sphingobacteriales* | | *Flexibacteraceae* | *Dyadobacter* |
|  |  | 788 | *Verrucomicrobia* | *Spartobacteria* | *Spartobacteriales* | | *Spartobacteriaceae* | MC18 |
|  |  | 789 | *Actinobacteria* | *Actinobacteria* | *Solirubrobacterales* | | *unclassified* | unclassified |
|  |  | 2064 | *Verrucomicrobia* | *Verrucomicrobiae* | *Verrucomicrobiales* | | *Verrucomicrobiaceae* | *Prosthecobacter* |
| H | | 34 | *AD3* | ABS-6 | unclassified | | unclassified | unclassified |
|  |  | 37 | *Actinobacteria* | *Actinobacteria* | *Solirubrobacterales* | | unclassified | unclassified |
|  |  | 51 | *Proteobacteria* | *Alphaproteobacteria* | unclassified | | unclassified | unclassified |
|  |  | 55 | *Acidobacteria* | iii1-8 | DS-18 | | unclassified | unclassified |
|  |  | 76 | *Actinobacteria* | *Actinobacteria* | *Actinomycetales* | | *Nocardioidaceae* | *Nocardioides* |
|  |  | 82 | *Proteobacteria* | *Deltaproteobacteria* | MIZ46 | | unclassified | unclassified |
|  |  | 92 | *Proteobacteria* | *Deltaproteobacteria* | *Myxococcales* | | *Polyangiaceae* | *Sorangium* |
|  |  | 107 | *Firmicutes* | *Bacilli* | *Bacillales* | | *Paenibacillaceae* | unclassified |
|  |  | 113 | *Proteobacteria* | *Betaproteobacteria* | *unclassified* | | unclassified | unclassified |
|  |  | 114 | *Nitrospirae* | *Nitrospira* | *Nitrospirales* | | FW | 4-29 |
|  |  | 117 | *Proteobacteria* | *Deltaproteobacteria* | *Myxococcales* | | unclassified | unclassified |
|  |  | 199 | *Proteobacteria* | *Gammaproteobacteria* | unclassified | | unclassified | unclassified |
|  |  | 229 | SM2F11 | unclassified | unclassified | | unclassified | unclassified |
|  |  | 263 | *Proteobacteria* | unclassified | unclassified | | unclassified | unclassified |
|  |  | 313 | *Proteobacteria* | *Gammaproteobacteria* | unclassified | | unclassified | unclassified |
|  |  | 385 | *Acidobacteria* | *Chloracidobacteria* | unclassified | | unclassified | unclassified |
|  |  | 423 | *Verrucomicrobia* | *Spartobacteria* | *Spartobacteriales* | | *Spartobacteriaceae* | *Chthoniobacter* |
|  |  | 466 | *Actinobacteria* | *Actinobacteria* | *Actinomycetales* | | *Actinosynnemataceae* | unclassified |
|  |  | 506 | *Verrucomicrobia* | *Opitutae* | *Opitutales* | | *Opitutaceae* | *Opitutus* |
|  |  | 520 | *Proteobacteria* | *Alphaproteobacteria* | unclassified | | unclassified | unclassified |
|  |  | 521 | *Proteobacteria* | *Alphaproteobacteria* | *Rhodospirillales* | | *Acetobacteraceae* | unclassified |
|  |  | 538 | *Acidobacteria* | *Acidobacteria* | *Acidobacteriales* | | unclassified | unclassified |
|  |  | 564 | WS3 | PRR-12 | MSB-4E2 | | unclassified | unclassified |
|  |  | 570 | *Chloroflexi* | Bljii12 | B07_WMSP1 | | FFCH4570 | unclassified |
|  |  | 576 | *Acidobacteria* | *Solibacteres* | *Solibacterales* | | *Solibacteraceae* | *Ca. Solibacter* |
|  |  | 581 | *Proteobacteria* | *Alphaproteobacteria* | *Rhizobiales* | | unclassified | unclassified |
|  |  | 590 | *Chloroflexi* | *Ktedonobacteria* | unclassified | | unclassified | unclassified |
|  |  | 661 | *Proteobacteria* | unclassified | unclassified | | unclassified | unclassified |
|  |  | 702 | *Chloroflexi* | SOGA31 | unclassified | | unclassified | unclassified |
|  |  | 728 | *Actinobacteria* | *Actinobacteria* | *Actinomycetales* | | unclassified | unclassified |
|  |  | 778 | *Actinobacteria* | *Actinobacteria* | *Acidimicrobiales* | | unclassified | unclassified |
| I* | | 6* | *Acidobacteria* | *Acidobacteria* | *Acidobacteriales* | | *Acidobacteriaceae* | unclassified |
|  |  | 7 | *Firmicutes* | *Bacilli* | *Bacillales* | | *Bacillaceae* | *Bacillus* |
|  |  | 15 | *Acidobacteria* | *Chloracidobacteria* | unclassified | | unclassified | unclassified |
|  |  | 57 | *Proteobacteria* | *Gammaproteobacteria* | unclassified | | unclassified | unclassified |
|  |  | 59 | *Firmicutes* | *Clostridia* | *Clostridiales* | | *Clostridiaceae* | Clostridium |
|  |  | 87 | *Gemmatimonadetes* | *Gemmatimonadete* | *Gemmatimonadales* | | unclassified | unclassified |
|  |  | 109 | *Elusimicrobia* | *Elusimicrobia* | FAC88 | | unclassified | unclassified |
|  |  | 150 | *Acidobacteria* | *Acidobacteria* | *Acidobacteriales* | | *Acidobacteriaceae* | unclassified |
|  |  | 151 | *Acidobacteria* | *Holophagae* | *Holophagales* | | *Holophagaceae* | unclassified |
|  |  | 163 | *Firmicutes* | *Clostridia* | *Clostridiales* | | *Clostridiaceae* | Clostridium |
|  |  | 178 | *Proteobacteria* | *Deltaproteobacteria* | *Syntrophobacterales* | | *Syntrophobacteraceae* | unclassified |
|  |  | 237* | *Planctomycetes* | *Phycisphaerae* | *Phycisphaerales* | | unclassified | unclassified |
|  |  | 271 | *Acidobacteria* | *Chloracidobacteria* | unclassified | | unclassified | unclassified |
|  |  | 280 | *Firmicutes* | *Clostridia* | *Clostridiales* | | *Clostridiaceae* | *Clostridium* |
|  |  | 355 | *Actinobacteria* | *Actinobacteria* | 0319-7L14 | | unclassified | unclassified |
|  |  | 412 | *Proteobacteria* | unclassified | unclassified | | unclassified | unclassified |
|  |  | 413 | *Acidobacteria* | iii1-8 | SJA-36 | | unclassified | unclassified |
|  |  | 417 | *Acidobacteria* | *Acidobacteria* | *Acidobacteriales* | | *Acidobacteriaceae* | unclassified |
|  |  | 435 | *Bacteroidetes* | *Sphingobacteria* | *Sphingobacteriales* | | unclassified | unclassified |
|  |  | 523 | *Bacteroidetes* | *Sphingobacteria* | *Sphingobacteriales* | | *Flexibacteraceae* | *Cytophaga* |
|  |  | 537 | *Actinobacteria* | *Actinobacteria* | *Acidimicrobiales* | | EB1017 | unclassified |
|  |  | 635 | *Firmicutes* | *Bacilli* | *Bacillales* | | unclassified | unclassified |
|  |  | 653 | *Bacteroidetes* | *Sphingobacteria* | *Sphingobacteriales* | | *Sphingobacteriaceae* | unclassified |
|  |  | 658* | *Proteobacteria* | *Betaproteobacteria* | *Burkholderiales* | | unclassified | unclassified |
|  |  | 672 | SPAM | FFCH6980 | unclassified | | unclassified | unclassified |
|  |  | 676 | *Firmicutes* | *Clostridia* | *Clostridiales* | | *Clostridiaceae* | *Clostridium* |
|  |  | 705 | *Firmicutes* | *Clostridia* | *Clostridiales* | | *Clostridiaceae* | *Clostridium* |
|  |  | 712 | *Bacteroidetes* | unclassified | unclassified | | unclassified | unclassified |
|  |  | 736 | *Proteobacteria* | *Alphaproteobacteria* | *Rhizobiales* | | unclassified | unclassified |
|  |  | 749 | *Acidobacteria* | iii1-8 | DS-18 | | unclassified | unclassified |
|  |  | 2269 | *Nitrospirae* | *Nitrospira* | *Nitrospirales* | | *Nitrospiraceae* | *Nitrospira* |
| RHIZOSPHERE | | | | | | | | |
| J | 23 | | *Nitrospirae* | *Nitrospira* | | *Nitrospirales* | *Nitrospiraceae* | *Nitrospira* |
|  | 41 | | *Proteobacteria* | *Betaproteobacteria* | | *Burkholderiales* | *Comamonadaceae* | unclassified |
|  | 56 | | *Chloroflexi* | *Chloroflexi* | | *Roseiflexales* | *Kouleothrixaceae* | unclassified |
|  | 73 | | *Bacteroidetes* | *Sphingobacteria* | | *Sphingobacteriales* | *Flexibacteraceae* | *Cytophaga* |
|  | 93 | | *Acidobacteria* | iii1-8 | | 32-20 | unclassified | unclassified |
|  | 126 | | *Bacteroidetes* | *Sphingobacteria* | | *Sphingobacteriales* | *Sphingobacteriaceae* | unclassified |
|  | 142 | | *Bacteroidetes* | *Flavobacteria* | | *Flavobacteriales* | *Cryomorphaceae* | *Fluviicola* |
|  | 151 | | *Acidobacteria* | *Holophagae* | | *Holophagales* | *Holophagaceae* | unclassified |
|  | 223 | | *Bacteroidetes* | *Sphingobacteria* | | *Sphingobacteriales* | *Flexibacteraceae* | *Dyadobacter* |
|  | 241 | | *Actinobacteria* | *Actinobacteria* | | *Solirubrobacterales* | *Patulibacteraceae* | unclassified |
|  | 282 | | *Chloroflexi* | Bljii12 | | B07_WMSP1 | FFCH4570 | unclassified |
|  | 336 | | *Actinobacteria* | *Actinobacteria* | | *Actinomycetales* | *Microbacteriaceae* | *Yonghaparkia* |
|  | 363 | | *Planctomycetes* | vadinHA49 | | unclassified | unclassified | unclassified |
|  | 464 | | *Elusimicrobia* | Elusimicrobia | | FAC88 | unclassified | unclassified |
|  | 478 | | *Verrucomicrobia* | *Verrucomicrobiae* | | *Verrucomicrobiales* | *Verrucomicrobia* sd. 3 | unclassified |
|  | 486 | | *Verrucomicrobia* | *Verrucomicrobiae* | | *Verrucomicrobiales* | unclassified | unclassified |
|  | 618 | | *Proteobacteria* | *Gammaproteobacteria* | | *Legionellales* | *Coxiellaceae* | *Aquicella* |
|  | 658 | | *Proteobacteria* | *Betaproteobacteria* | | *Burkholderiales* | unclassified | unclassified |
|  | 661 | | *Proteobacteria* | unclassified | | unclassified | unclassified | unclassified |
|  | 771 | | *Actinobacteria* | *Actinobacteria* | | unclassified | unclassified | unclassified |
|  | 782 | | *Proteobacteria* | *Alphaproteobacteria* | | *Sphingomonadales* | *Sphingomonadaceae* | unclassified |
|  | 2269 | | *Nitrospirae* | *Nitrospira* | | *Nitrospirales* | *Nitrospiraceae* | *Nitrospira* |
|  | 2292 | | *Bacteroidetes* | *Sphingobacteria* | | *Sphingobacteriales* | unclassified | unclassified |
| K* | 2 | | *Planctomycetes* | unclassified | | unclassified | unclassified | unclassified |
|  | 62 | | *Verrucomicrobia* | *Opitutae* | | *Opitutales* | *Opitutaceae* | *Opitutus* |
|  | 68 | | *Bacteroidetes* | *Flavobacteria* | | *Flavobacteriales* | *Flavobacteriaceae* | *Flavobacterium* |
|  | 112 | | WPS-2 | unclassified | | unclassified | unclassified | unclassified |
|  | 114 | | *Nitrospirae* | *Nitrospira* | | *Nitrospirales* | FW | 4-29 |
|  | 132 | | *Acidobacteria* | *Solibacteres* | | *Solibacterales* | *Solibacteraceae* | *Ca. Solibacter* |
|  | 154 | | *Gemmatimonadetes* | *Gemmatimonadetes* | | *Gemmatimonadales* | *Gemmatimonadaceae* | *Gemmatimonas* |
|  | 161* | | *Actinobacteria* | *Actinobacteria* | | *Actinomycetales* | *Streptomycetaceae* | *Streptacidiphilus* |
|  | 200 | | *Acidobacteria* | *Acidobacteria* | | *Acidobacteriales* | *Acidobacteriaceae* | unclassified |
|  | 316* | | *Actinobacteria* | *Actinobacteria* | | *Acidimicrobiales* | EB1017 | unclassified |
|  | 328 | | *Acidobacteria* | PAUC37f | | unclassified | unclassified | unclassified |
|  | 466 | | *Actinobacteria* | *Actinobacteria* | | *Actinomycetales* | *Actinosynnemataceae* | unclassified |
|  | 469 | | *Actinobacteria* | *Actinobacteria* | | *Actinomycetales* | unclassified | unclassified |
|  | 498 | | *Chloroflexi* | *Anaerolineae* | | H39 | unclassified | unclassified |
|  | 505 | | *Verrucomicrobia* | *Opitutae* | | *Opitutales* | *Opitutaceae* | *Opitutus* |
|  | 556 | | *Chloroflexi* | *Anaerolineae* | | *Caldilineales* | *Caldilineaceae* | *Caldilinea* |
|  | 592 | | *Proteobacteria* | *Gammaproteobacteria* | | unclassified | unclassified | unclassified |
|  | 682 | | *Actinobacteria* | *Actinobacteria* | | MC47 | unclassified | unclassified |
| L* | 37 | | *Actinobacteria* | *Actinobacteria* | | *Solirubrobacterales* | unclassified | unclassified |
|  | 45 | | *Proteobacteria* | *Gammaproteobacteria* | | *Enterobacteriales* | *Enterobacteriaceae* | unclassified |
|  | 82 | | *Proteobacteria* | *Deltaproteobacteria* | | MIZ46 | unclassified | unclassified |
|  | 105 | | *Bacteroidetes* | *Sphingobacteria* | | *Sphingobacteriales* | unclassified | unclassified |
|  | 242 | | *Verrucomicrobia* | *Methylacidiphilae* | | *Methylacidiphilales* | LD19 | unclassified |
|  | 255 | | *Proteobacteria* | *Betaproteobacteria* | | *Burkholderiales* | *Burkholderiaceae* | *Burkholderia* |
|  | 355 | | *Actinobacteria* | *Actinobacteria* | | 0319-7L14 | unclassified | unclassified |
|  | 359 | | *Proteobacteria* | *Betaproteobacteria* | | *Burkholderiales* | *Burkholderiaceae* | *Burkholderia* |
|  | 400 | | *Chloroflexi* | SOGA31 | | unclassified | unclassified | unclassified |
|  | 404 | | *Actinobacteria* | *Actinobacteria* | | *Actinomycetales* | *Thermomonosporaceae* | unclassified |
|  | 481* | | *Actinobacteria* | *Actinobacteria* | | *Actinomycetales* | unclassified | unclassified |
|  | 563 | | *Actinobacteria* | *Actinobacteria* | | *Actinomycetales* | unclassified | unclassified |
|  | 612 | | *Proteobacteria* | *Deltaproteobacteria* | | *Bdellovibrionales* | *Bdellovibrionaceae* | *Bdellovibrio* |
|  | 655 | | *Verrucomicrobia* | *Verrucomicrobiae* | | *Verrucomicrobiales* | *Verrucomicrobiaceae* | *Prosthecobacter* |
|  | 681 | | *Acidobacteria* | *Acidobacteria* | | *Acidobacteriales* | *Acidobacteriaceae* | unclassified |
|  | 694 | | *Actinobacteria* | *Actinobacteria* | | *Acidimicrobiales* | CL500-29 | unclassified |
|  | 701 | | *Acidobacteria* | *Acidobacteria* | | *Acidobacteriales* | unclassified | unclassified |
| M | 64 | | *Elusimicrobia* | *Elusimicrobia* | | *FAC88* | unclassified | unclassified |
|  | 104 | | *Bacteroidetes* | *Flavobacteria* | | *Flavobacteriales* | *Flavobacteriaceae* | *Chryseobacterium* |
|  | 191 | | *Acidobacteria* | iii1-8 | | 32-20 | unclassified | unclassified |
|  | 228 | | *Armatimonadetes* | unclassified | | unclassified | unclassified | unclassified |
|  | 327 | | *Actinobacteria* | *Actinobacteria* | | *Actinomycetales* | *Actinospicaceae* | unclassified |
|  | 399 | | *Proteobacteria* | *Gammaproteobacteria* | | *Xanthomonadales* | *Xanthomonadaceae* | *Rhodanobacter* |
|  | 473 | | *Proteobacteria* | *Alphaproteobacteria* | | *Sphingomonadales* | *Sphingomonadaceae* | *Novosphingobium* |
|  | 497 | | *Chloroflexi* | *Anaerolineae* | | BPC110 | unclassified | unclassified |
|  | 510 | | *Proteobacteria* | *Gammaproteobacteria* | | *Pseudomonadales* | *Pseudomonadaceae* | *Pseudomonas* |
|  | 576 | | *Acidobacteria* | *Solibacteres* | | *Solibacterales* | *Solibacteraceae* | *Ca. Solibacter* |
|  | 581 | | *Proteobacteria* | *Alphaproteobacteria* | | *Rhizobiales* | unclassified | unclassified |
|  | 657 | | *Proteobacteria* | *Gammaproteobacteria* | | *Pseudomonadales* | *Pseudomonadaceae* | Pseudomonas |
|  | 710 | | *Actinobacteria* | *Actinobacteria* | | *Actinomycetales* | *Geodermatophilaceae* | *Geodermatophilus* |
|  | 749 | | *Acidobacteria* | iii1-8 | | DS-18 | unclassified | unclassified |
|  | 751 | | *Acidobacteria* | iii1-8 | | 32-20 | unclassified | unclassified |
|  | 794 | | *Proteobacteria* | *Deltaproteobacteria* | | *Bdellovibrionales* | *Bdellovibrionaceae* | *Bdellovibrio* |
| N | 43 | | *Bacteroidetes* | *Sphingobacteria* | | *Sphingobacteriales* | unclassified | unclassified |
|  | 69 | | *Acidobacteria* | *Solibacteres* | | *Solibacterales* | *Solibacteraceae* | *Ca. Solibacter* |
|  | 175 | | *Proteobacteria* | *Betaproteobacteria* | | *Burkholderiales* | *Oxalobacteraceae* | *Collimonas* |
|  | 233 | | *Bacteroidetes* | *Flavobacteria* | | *Flavobacteriales* | *Flavobacteriaceae* | unclassified |
|  | 341 | | *Verrucomicrobia* | *Verrucomicrobiae* | | *Verrucomicrobiales* | *Verrucomicrobiaceae* | *Luteolibacter* |
|  | 371 | | *Proteobacteria* | unclassified | | unclassified | unclassified | unclassified |
|  | 602 | | *Gemmatimonadetes* | *Gemmatimonadetes* | | *Gemmatimonadales* | unclassified | unclassified |
|  | 653 | | *Bacteroidetes* | *Sphingobacteria* | | *Sphingobacteriales* | *Sphingobacteriaceae* | unclassified |
|  | 728 | | *Actinobacteria* | *Actinobacteria* | | *Actinomycetales* | unclassified | unclassified |
|  | 2064 | | *Verrucomicrobia* | *Verrucomicrobiae* | | *Verrucomicrobiales* | *Verrucomicrobiaceae* | *Prosthecobacter* |
| O* | 7 | | *Firmicutes* | *Bacilli* | | *Bacillales* | *Bacillaceae* | Bacillus |
|  | 33 | | *Proteobacteria* | *Alphaproteobacteria* | | *Rhodospirillales* | *Acetobacteraceae* | unclassified |
|  | 79 | | *Acidobacteria* | *Solibacteres* | | *Solibacterales* | *Solibacteraceae* | *Ca. Solibacter* |
|  | 94 | | *Acidobacteria* | *Solibacteres* | | *Solibacterales* | *Solibacteraceae* | *Ca. Solibacter* |
|  | 125 | | *Bacteroidetes* | *Sphingobacteria* | | *Sphingobacteriales* | *Flexibacteraceae* | *Cytophaga* |
|  | 133 | | *Acidobacteria* | unclassified | | unclassified | unclassified | unclassified |
|  | 163* | | *Firmicutes* | *Clostridia* | | *Clostridiales* | *Clostridiaceae* | *Clostridium* |
|  | 171 | | *Verrucomicrobia* | *Spartobacteria* | | *Spartobacteriales* | *Spartobacteriaceae* | MC18 |
|  | 173* | | *Planctomycetes* | agg27 | | OM190 | unclassified | unclassified |
|  | 257 | | *Firmicutes* | *Clostridia* | | *Clostridiales* | *Clostridiaceae* | *Caloramator* |
|  | 263 | | *Proteobacteria* | unclassified | | unclassified | unclassified | unclassified |
|  | 310 | | *Acidobacteria* | *Solibacteres* | | *Solibacterales* | *Solibacteraceae* | *Ca. Solibacter* |
|  | 322 | | *Chloroflexi* | SOGA31 | | unclassified | unclassified | unclassified |
|  | 331 | | *Actinobacteria* | *Actinobacteria* | | unclassified | unclassified | unclassified |
|  | 388 | | *Acidobacteria* | *Chloracidobacteria* | | unclassified | unclassified | unclassified |
|  | 541 | | *Actinobacteria* | *Actinobacteria* | | *Actinomycetales* | *Actinospicaceae* | unclassified |
|  | 603 | | *Acidobacteria* | *Solibacteres* | | *Solibacterales* | *Solibacteraceae* | *Ca. Solibacter* |
|  | 607 | | *Proteobacteria* | *Alphaproteobacteria* | | *Rhodospirillales* | *Acetobacteraceae* | unclassified |
|  | 654 | | *Chloroflexi* | *Anaerolineae* | | SJA-15 | unclassified | unclassified |
|  | 712 | | *Bacteroidetes* | unclassified | | unclassified | unclassified | unclassified |
| P | 12 | | *Actinobacteria* | *Actinobacteria* | | *Acidimicrobiales* | *Microthrixaceae* | unclassified |
|  | 32 | | *Acidobacteria* | *Chloracidobacteria* | | *unclassified* | unclassified | unclassified |
|  | 48 | | *Verrucomicrobia* | *Spartobacteria* | | *Spartobacteriales* | *Spartobacteriaceae* | unclassified |
|  | 61 | | *Actinobacteria* | *Actinobacteria* | | *Acidimicrobiales* | AKIW874 | unclassified |
|  | 107 | | *Firmicutes* | *Bacilli* | | *Bacillales* | *Paenibacillaceae* | unclassified |
|  | 108 | | *Actinobacteria* | *Actinobacteria* | | *Actinomycetales* | *Microbacteriaceae* | unclassified |
|  | 120 | | Planctomycetes | agg27 | | CL500-15 | unclassified | unclassified |
|  | 138 | | Planctomycetes | unclassified | | unclassified | unclassified | unclassified |
|  | 170 | | *Proteobacteria* | *Alphaproteobacteria* | | *Rickettsiales* | *Rickettsiaceae* | *Rickettsia* |
|  | 179 | | *Actinobacteria* | *Actinobacteria* | | *Actinomycetales* | *Streptomycetaceae* | *Streptomyces* |
|  | 183 | | *Planctomycetes* | *Phycisphaerae* | | unclassified | unclassified | unclassified |
|  | 217 | | *Planctomycetes* | *Planctomycea* | | *Gemmatales* | *Gemmataceae* | unclassified |
|  | 261 | | *Chloroflexi* | TK17 | | S085 | unclassified | unclassified |
|  | 268 | | *Verrucomicrobia* | unclassified | | unclassified | unclassified | unclassified |
|  | 275 | | TM7 | TM7-1 | | unclassified | unclassified | unclassified |
|  | 276 | | *Armatimonadetes* | *Armatimonadia* | | *Armatimonadales* | WD294 | unclassified |
|  | 302 | | *Proteobacteria* | *Alphaproteobacteria* | | *Rhizobiales* | *Hyphomicrobiaceae* | *Hyphomicrobium* |
|  | 326 | | *Actinobacteria* | *Actinobacteria* | | *Actinomycetales* | *Microbacteriaceae* | *Microbacterium* |
|  | 391 | | *Chlamydiae* | *Chlamydiae* | | *Chlamydiales* | *Parachlamydiaceae* | *Ca. Protochlamydia* |
|  | 397 | | *Proteobacteria* | *Gammaproteobacteria* | | *Legionellales* | *Coxiellaceae* | *Aquicella* |
|  | 538 | | *Acidobacteria* | *Acidobacteria* | | *Acidobacteriales* | unclassified | unclassified |
|  | 572 | | *Actinobacteria* | *Actinobacteria* | | *Acidimicrobiales* | unclassified | unclassified |
|  | 611 | | *Actinobacteria* | *Actinobacteria* | | *Actinomycetales* | unclassified | unclassified |
|  | 642 | | *Gemmatimonadetes* | *Gemmatimonadetes* | | *Gemmatimonadales* | *Gemmatimonadaceae* | *Gemmatimonas* |
|  | 668 | | *Chlorobi* | BSV19 | | unclassified | unclassified | unclassified |
|  | 722 | | *Actinobacteria* | *Actinobacteria* | | *Actinomycetales* | *Streptomycetaceae* | *Streptomyces* |
|  | 740 | | *Proteobacteria* | *Gammaproteobacteria* | | *Xanthomonadales* | *Xanthomonadaceae* | *Arenimonas* |
|  | 778 | | *Actinobacteria* | *Actinobacteria* | | *Acidimicrobiales* | unclassified | unclassified |
| Q* | 1 | | *Proteobacteria* | unclassified | | unclassified | unclassified | unclassified |
|  | 14 | | *Proteobacteria* | *Deltaproteobacteria* | | *Bdellovibrionales* | *Bdellovibrionaceae* | *Bdellovibrio* |
|  | 44 | | TM7 | unclassified | | unclassified | unclassified | unclassified |
|  | 52 | | *Acidobacteria* | unclassified | | unclassified | unclassified | unclassified |
|  | 55 | | *Acidobacteria* | iii1-8 | | DS-18 | unclassified | unclassified |
|  | 67 | | *Bacteroidetes* | *Sphingobacteria* | | *Sphingobacteriales* | unclassified | unclassified |
|  | 92 | | *Proteobacteria* | *Deltaproteobacteria* | | *Myxococcales* | *Polyangiaceae* | *Sorangium* |
|  | 109 | | *Elusimicrobia* | *Elusimicrobia* | | FAC88 | unclassified | unclassified |
|  | 123 | | *Actinobacteria* | *Actinobacteria* | | *Actinomycetales* | *Cellulomonadaceae* | Demequina |
|  | 128 | | *Proteobacteria* | *Betaproteobacteria* | | *Burkholderiales* | *Burkholderiaceae* | Cupriavidus |
|  | 150 | | *Acidobacteria* | *Acidobacteria* | | *Acidobacteriales* | *Acidobacteriaceae* | unclassified |
|  | 178 | | *Proteobacteria* | *Deltaproteobacteria* | | *Syntrophobacterales* | *Syntrophobacteraceae* | unclassified |
|  | 190 | | *Acidobacteria* | *Acidobacteria* | | *Acidobacteriales* | unclassified | unclassified |
|  | 204 | | *Chloroflexi* | SOGA31 | | unclassified | unclassified | unclassified |
|  | 211 | | *Planctomycetes* | vadinHA49 | | unclassified | unclassified | unclassified |
|  | 227* | | *Proteobacteria* | unclassified | | unclassified | unclassified | unclassified |
|  | 271 | | *Acidobacteria* | *Chloracidobacteria* | | unclassified | unclassified | unclassified |
|  | 305 | | *Proteobacteria* | unclassified | | unclassified | unclassified | unclassified |
|  | 344 | | *Actinobacteria* | *Actinobacteria* | | *Actinomycetales* | *Micrococcaceae* | *Arthrobacter* |
|  | 356 | | *Bacteroidetes* | unclassified | | unclassified | unclassified | unclassified |
|  | 398* | | *Spirochaetes* | *Leptospirae* | | *Leptospirales* | *Leptospiraceae* | unclassified |
|  | 413 | | *Acidobacteria* | iii1-8 | | SJA-36 | unclassified | unclassified |
|  | 435 | | *Bacteroidetes* | *Sphingobacteria* | | *Sphingobacteriales* | unclassified | unclassified |
|  | 446 | | *Chloroflexi* | *Chloroflexi* | | *Roseiflexales* | *Kouleothrixaceae* | unclassified |
|  | 460 | | *Actinobacteria* | *Actinobacteria* | | MC47 | unclassified | unclassified |
|  | 506 | | *Verrucomicrobia* | *Opitutae* | | *Opitutales* | *Opitutaceae* | *Opitutus* |
|  | 520* | | *Proteobacteria* | *Alphaproteobacteria* | | unclassified | unclassified | unclassified |
|  | 545 | | *Acidobacteria* | *Chloracidobacteria* | | unclassified | unclassified | unclassified |
|  | 551 | | *Bacteroidetes* | *Sphingobacteria* | | *Sphingobacteriales* | unclassified | unclassified |
|  | 578 | | *Acidobacteria* | *Chloracidobacteria* | | unclassified | unclassified | unclassified |
|  | 639 | | *Proteobacteria* | *Alphaproteobacteria* | | unclassified | unclassified | unclassified |
|  | 667 | | *Proteobacteria* | *Gammaproteobacteria* | | *Enterobacteriales* | *Enterobacteriaceae* | unclassified |
|  | 670 | | *Actinobacteria* | *Actinobacteria* | | *Actinomycetales* | unclassified | unclassified |
|  | 674 | | *Proteobacteria* | *Betaproteobacteria* | | unclassified | unclassified | unclassified |
|  | 678 | | *Proteobacteria* | *Gammaproteobacteria* | | unclassified | unclassified | unclassified |
|  | 702 | | *Chloroflexi* | SOGA31 | | unclassified | unclassified | unclassified |
|  | 708 | | *Elusimicrobia* | *Elusimicrobia* | | *Elusimicrobiales* | unclassified | unclassified |
| R | 10 | | *Proteobacteria* | *Betaproteobacteria* | | unclassified | unclassified | unclassified |
|  | 18 | | *Proteobacteria* | *Deltaproteobacteria* | | *Myxococcales* | *Haliangiaceae* | unclassified |
|  | 22 | | *Actinobacteria* | *Actinobacteria* | | *Actinomycetales* | *Nakamurellaceae* | unclassified |
|  | 65 | | TM6 | SJA-4 | | unclassified | unclassified | unclassified |
|  | 85 | | *Proteobacteria* | *Alphaproteobacteria* | | *Rhizobiales* | *Hyphomicrobiaceae* | unclassified |
|  | 90 | | *Firmicutes* | *Bacilli* | | *Bacillales* | unclassified | unclassified |
|  | 116 | | *Acidobacteria* | *Acidobacteria* | | *Acidobacteriales* | unclassified | unclassified |
|  | 119 | | *Actinobacteria* | *Actinobacteria* | | *Actinomycetales* | *Intrasporangiaceae* | *Terracoccus* |
|  | 156 | | *Proteobacteria* | *Betaproteobacteria* | | unclassified | unclassified | unclassified |
|  | 180 | | *Chloroflexi* | *Anaerolineae* | | unclassified | unclassified | unclassified |
|  | 193 | | *Actinobacteria* | *Actinobacteria* | | 0319-7L14 | unclassified | unclassified |
|  | 265 | | *Actinobacteria* | *Actinobacteria* | | *Actinomycetales* | unclassified | unclassified |
|  | 297 | | *Acidobacteria* | *Acidobacteria* | | *Acidobacteriales* | *Acidobacteriaceae* | unclassified |
|  | 329 | | *Proteobacteria* | *Deltaproteobacteria* | | *Desulfuromonadales* | *Geobacteraceae* | *Geobacter* |
|  | 432 | | *Proteobacteria* | *Betaproteobacteria* | | unclassified | unclassified | unclassified |
|  | 522 | | *Actinobacteria* | *Actinobacteria* | | *Actinomycetales* | unclassified | unclassified |
|  | 532 | | *Actinobacteria* | *Actinobacteria* | | *Actinomycetales* | *Geodermatophilaceae* | *Geodermatophilus* |
|  | 544 | | *Proteobacteria* | *Betaproteobacteria* | | *Methylophilales* | Methylophilaceae | unclassified |
|  | 632 | | *Actinobacteria* | *Actinobacteria* | | *Actinomycetales* | Nocardioidaceae | unclassified |
|  | 646 | | *Actinobacteria* | *Actinobacteria* | | *Actinomycetales* | unclassified | unclassified |
|  | 659 | | *Elusimicrobia* | *Elusimicrobia* | | *FAC88* | unclassified | unclassified |
|  | 664 | | *Firmicutes* | *Bacilli* | | *Lactobacillales* | *Enterococcaceae* | *Enterococcus* |
|  | 669 | | *Actinobacteria* | *Actinobacteria* | | *Acidimicrobiales* | CL500-29 | unclassified |
| OTUs not involved in pMENs | 17* | | *Actinobacteria* | *Actinobacteria* | | *Actinomycetales* | *Micromonosporaceae* | *Verrucosispora* |
|  | 127* | | *Bacteroidetes* | *Bacteroidia* | | *Bacteroidales* | *Porphyromonadaceae* | *Dysgonomonas* |
|  | 258* | | *Actinobacteria* | *Actinobacteria* | | *Actinomycetales* | *Actinomycetaceae* | *Actinomyces* |
|  | 406* | | *Actinobacteria* | *Actinobacteria* | | *Actinomycetales* | *Gordoniaceae* | *Gordonia* |
|  | 696* | | *Acidobacteria* | *Acidobacteria* | | *Acidobacteriales* | *Acidobacteriaceae* | *Acidobacterium* |
|  | 741* | | *Verrucomicrobia* | *Verrucomicrobiae* | | *Verrucomicrobiales* | *Verrucomicrobiaceae* | *Luteolibacter* |

**Supplementary Table 6.** Classified bacterial genera (with respective phyla and OTUs) that proportions were significantly higher in humidified or control plots bulk soil or rhizosphere (or in both soil compartments) according to the LefSe analysis (LDA scores higher than 2.0). The OTUs numbers correspond to phylotypes numbers in Supplementary Table 4. The capital letters in superscripts indicate pMENs modules (first for bulk soil and second for rhizosphere) where the OTU was detected. The asterisks mark OTUs that were not found in respective soil compartment bacterial network`s modules. Abbreviations: SC, soil compartment; Treat., treatment; RP, ratio of proportions (humidified/control); *Ca.*, candidate genus.

| SC | Treat. | Phylum | Genus | OTUs | RP |
| --- | --- | --- | --- | --- | --- |
| Bulk soil | Humidified | *Acidobacteria* | *Ca. Solibacter* | 440^F,^* | 1.33 |
|  |  |  | *Gordonia* | 406*^,^* | 1.41 |
|  |  | *Proteobacteria* | *Aquicella* | 397^C,P^ | 2.33 |
|  |  |  | *Geobacter* | 49^C,^*, 329^C,R^ | 4.87 |
|  |  |  | *Pedomicrobium* | 368^C,^* | 1.40 |
|  | Control | *Actinobacteria* | *Actinomyces* | 258*^,^* | 0.01^1^ |
|  |  |  | *Microbacterium* | 11^B,^* | 0.36 |
|  |  |  | *Verrucosispora* | 17*^,^* | 0.50 |
|  |  | *Bacteroidetes* | *Dysgonomonas* | 127*^,^* | 0.14^1^ |
|  |  | *Firmicutes* | *Solibacillus* | 462^G,^* | 0.33 |
|  |  | *Gemmatimonadetes* | *Gemmatimonas* | 642*^,P^ | 0.43 |
|  |  | *Proteobacteria* | *Arenimonas* | 740*,^P^ | 0.47 |
|  |  |  | *Pseudomonas* | 657^G,M^ | 0.29 |
|  |  |  | *Rhodanobacter* | 399^G,M^ | 0.41 |
| Rhizosphere | Humidified | *Bactroidetes* | *Cytophaga* | 523^I,^* | 1.50 |
|  |  | *Firmicutes* | *Clostridium* | 163^I,O^ | 5.50 |
|  |  | *Proteobacteria* | *Geobacter* | 49^C,^*, 640^F,^*, 754^C,^* | 10.3 |
|  |  |  | *Hyphomicrobium* | 302*^,P^ | 2.00 |
|  |  | *Verrucomicrobia* | *Ca. Xiphinematobacter* | 84^A,^* | 2.60 |
|  |  |  | *Luteolibacter* | 741*^,^* | 2.25 |
|  |  |  | *MC18* | 788^G,^* | 2.00 |
|  |  |  | *Opititus* | 506^H,Q^ | 2.00 |
|  | Control | *Acidobacteria* | *Acidobacterium* | 696*^,^* | 0.55 |
|  |  |  | *Ca. Solibacter* | 79*^,O^ | 0.25 |
|  |  | *Actinobacteria* | *Streptacidiphilus* | 161*^,K^ | 0.19 |
|  |  | *Proteobacteria* | *Pseudomonas* | 657^G,M^,779^F,^* | 0.20 |
|  |  |  | *Rhodanobacter* | 399^G,M^ | 0.70 |

^1^ Proportion of the phylotype in humidified plots bacterial community (the respective proportion in control plots was 0).

## Supplementary Figures


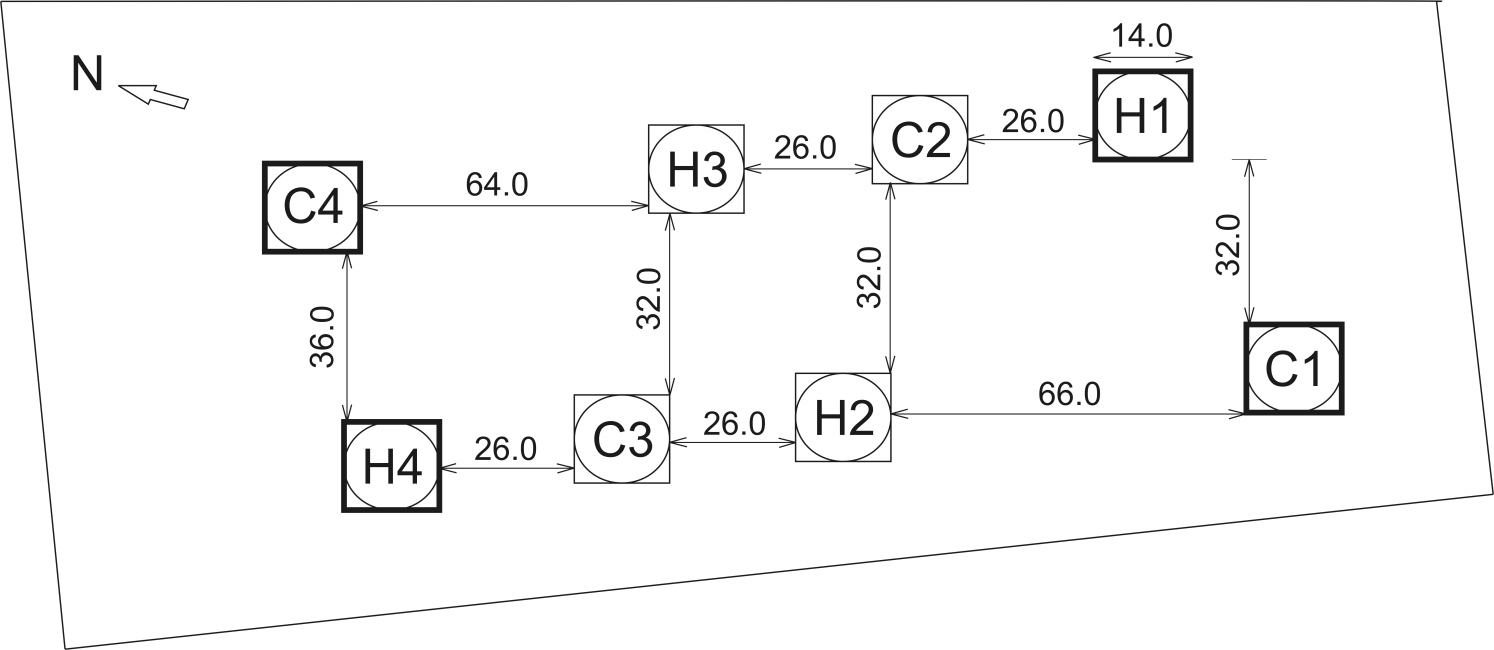


**Supplementary Figure 1.** The scheme of the experimental area. The analysed control and humidified plots (C and H, respectively) are indicated with bold frames. The numbers above the arrows indicate distances between plots (m).


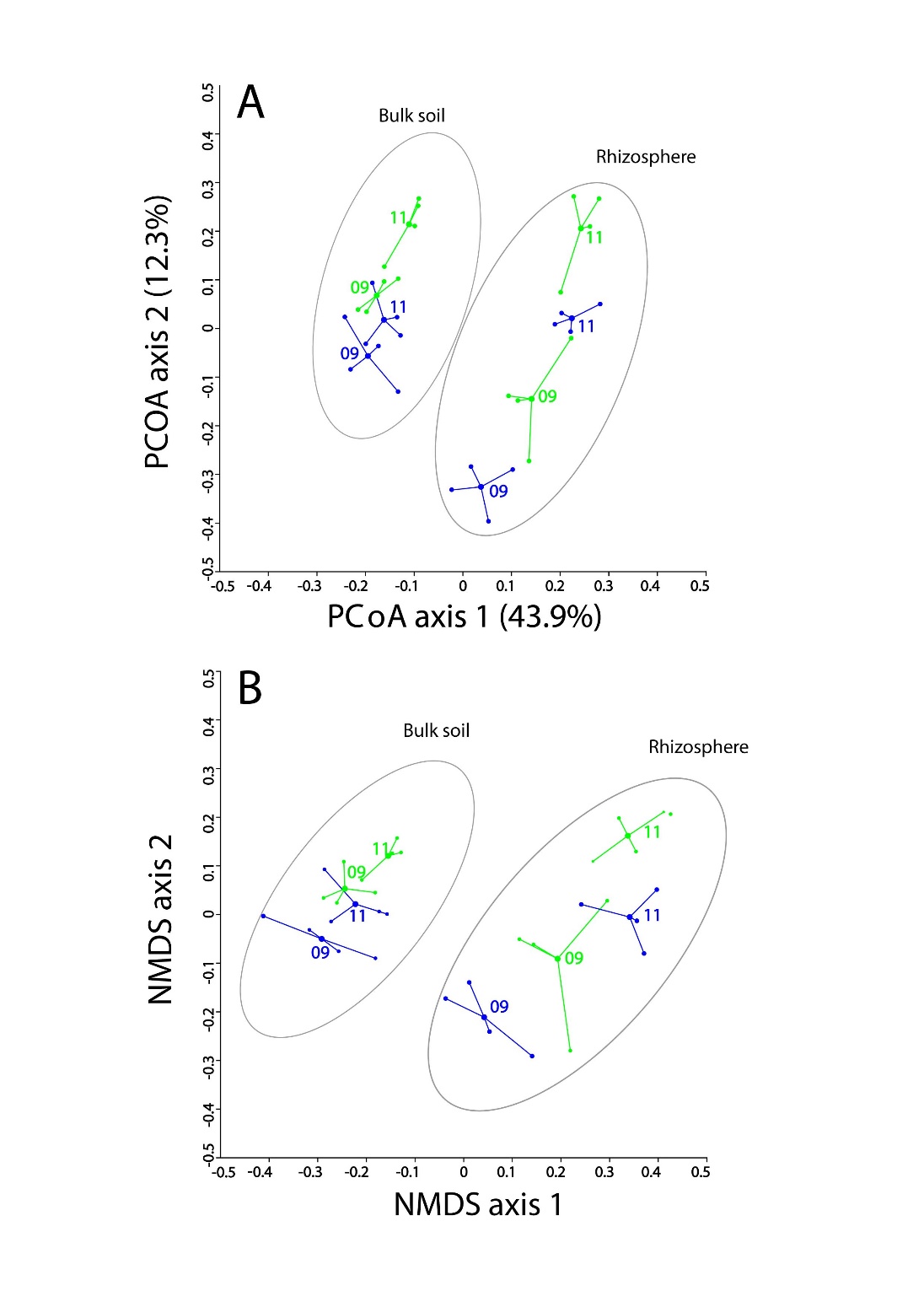


**Supplementary Figure 2.** Ordination of bulk soil and rhizosphere samples from control and humidified plots from years 2009 and 2011 according to principal coordinates analysis (PCoA) (A) and non-metrical multidimensional scaling (NMDS) (B) using Bray-Curtis dissimilarity matrix. Individual samples are connencted with lines to group centroid. Green circles indicate samples from control plots and blue circles indicate samples from humidified plots. Abbreaviations: 09 - 2009, 11- 2011.

**
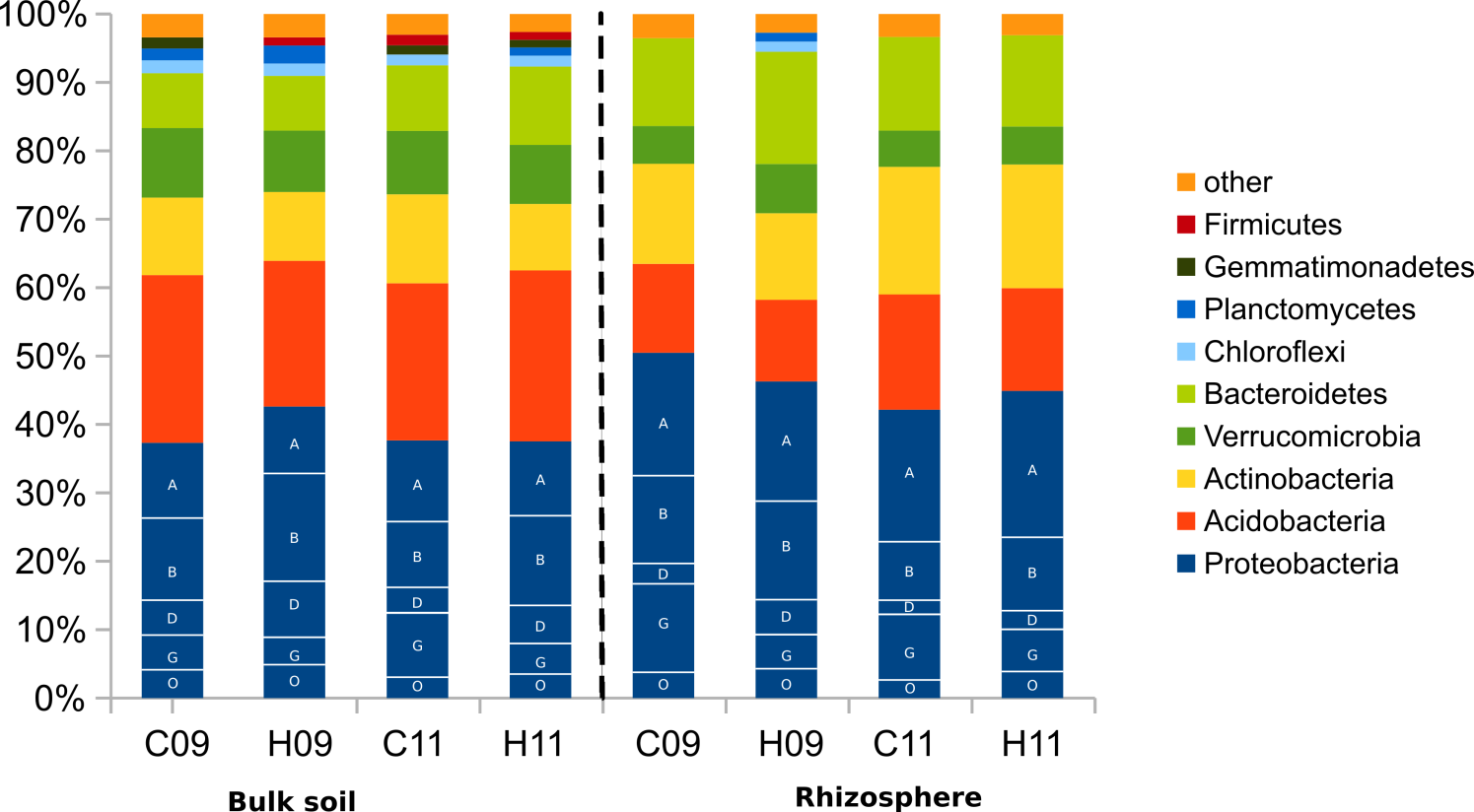
**

**Supplementary Figure 3.** Proportions of the dominating bacterial phyla and proteobacterial classes (A - Alfaproteobacteria, B – Betaproteobacteria, D – Deltaproteobacteria, G - Gammaproteobacteria, O-others) identified in each soil group. Percentages of the sequences belonging to the respective phyla and proteobacterial classes are shown. Others indicate a proportional sum of all phyla that were less represented than the 1% in the bacterial communities. C and H indicate control and humidified plots, respectively, and 09 and 11 indicate study years (2009 and 2011, respectively).


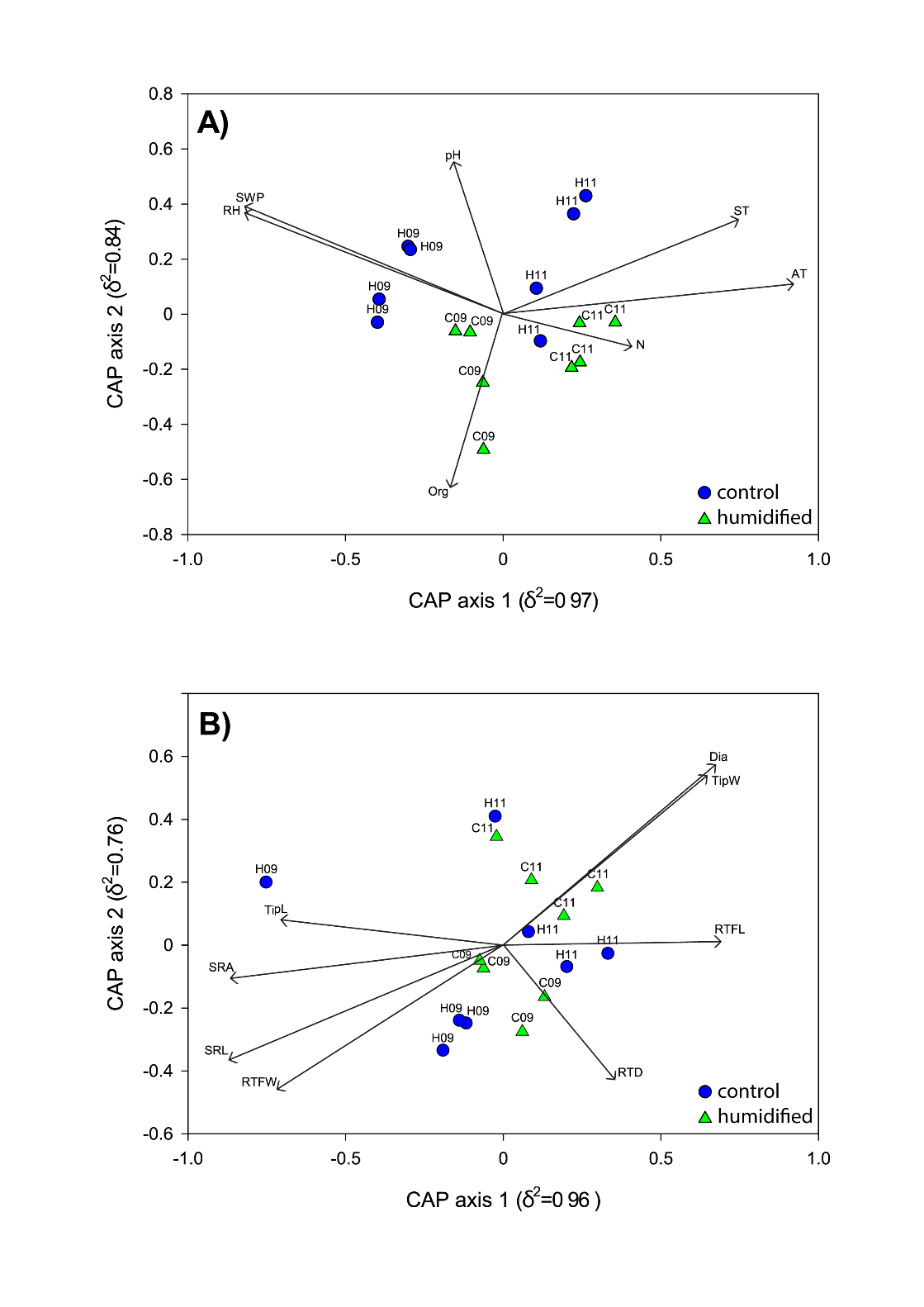


**Supplementary Figure 4.** Canonical Analysis of Principal Coordinates (CAP) ordination showing variation in bulk soil bacterial community composition among sites and strength and direction of correlations of constraining plot climatic and soil (A), and root (B) variables (arrows).


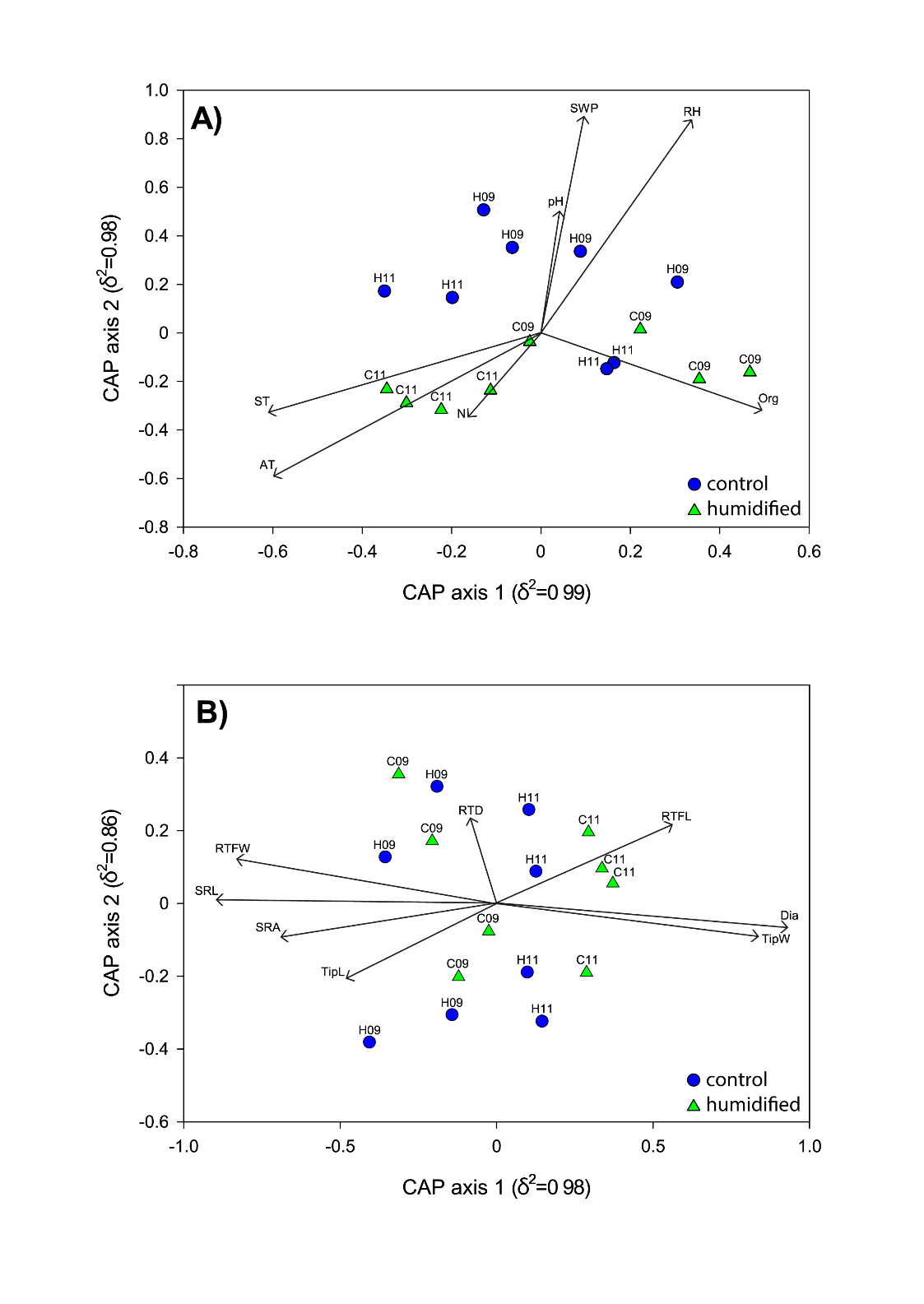


**Supplementary Figure 5.** Canonical Analysis of Principal Coordinates (CAP) ordination showing variation in rhizosphere soil bacterial community composition among sites and strength and direction of correlations of constraining plot climatic and soil (A), and root (B) variables (arrows).
